# Calpain activity is negatively regulated by a KCTD7–Cullin-3 complex via non-degradative ubiquitination

**DOI:** 10.1038/s41421-023-00533-3

**Published:** 2023-03-24

**Authors:** Jaiprakash Sharma, Shalaka Mulherkar, Uan-I Chen, Yan Xiong, Lakshya Bajaj, Byoung-Kyu Cho, Young Ah Goo, Hon-Chiu Eastwood Leung, Kimberley F. Tolias, Marco Sardiello

**Affiliations:** 1 Department of Molecular and Human Genetics, Baylor College of Medicine, Jan and Dan Duncan Neurological Research Institute, Texas Children’s Hospital, Houston, TX USA; 2 Department of Pediatrics, Washington University in St. Louis, School of Medicine, Genetics and Genomic Medicine, Saint Louis, MO USA; 3 Department of Neuroscience, Baylor College of Medicine, Houston, TX USA; 4 Mass Spectrometry Technology Access Center at the McDonnell Genome Institute, Washington University in St. Louis, School of Medicine, St. Louis, MO USA; 5 Department of Biochemistry and Molecular Biophysics, Department of Genetics, Washington University School of Medicine, St. Louis, MO USA; 6 Departments of Medicine, Pediatrics, and Molecular and Cellular Biology, Dan Duncan Cancer Center, Baylor College of Medicine, Houston, TX USA; 7 Verna and Marrs McLean Department of Biochemistry and Molecular Cell Biology, Baylor College of Medicine, Houston, TX USA

**Keywords:** Ubiquitylation, Ubiquitin ligases, Ubiquitylation, Mechanisms of disease

## Abstract

Calpains are a class of non-lysosomal cysteine proteases that exert their regulatory functions via limited proteolysis of their substrates. Similar to the lysosomal and proteasomal systems, calpain dysregulation is implicated in the pathogenesis of neurodegenerative disease and cancer. Despite intensive efforts placed on the identification of mechanisms that regulate calpains, however, calpain protein modifications that regulate calpain activity are incompletely understood. Here we show that calpains are regulated by KCTD7, a cytosolic protein of previously uncharacterized function whose pathogenic mutations result in epilepsy, progressive ataxia, and severe neurocognitive deterioration. We show that KCTD7 works in complex with Cullin-3 and Rbx1 to execute atypical, non-degradative ubiquitination of calpains at specific sites (K398 of calpain 1, and K280 and K674 of calpain 2). Experiments based on single-lysine mutants of ubiquitin determined that KCTD7 mediates ubiquitination of calpain 1 via K6-, K27-, K29-, and K63-linked chains, whereas it uses K6-mediated ubiquitination to modify calpain 2. Loss of KCTD7-mediated ubiquitination of calpains led to calpain hyperactivation, aberrant cleavage of downstream targets, and caspase-3 activation. CRISPR/Cas9-mediated knockout of *Kctd7* in mice phenotypically recapitulated human KCTD7 deficiency and resulted in calpain hyperactivation, behavioral impairments, and neurodegeneration. These phenotypes were largely prevented by pharmacological inhibition of calpains, thus demonstrating a major role of calpain dysregulation in KCTD7-associated disease. Finally, we determined that Cullin-3–KCTD7 mediates ubiquitination of all ubiquitous calpains. These results unveil a novel mechanism and potential target to restrain calpain activity in human disease and shed light on the molecular pathogenesis of KCTD7-associated disease.

## Introduction

Calpains are a unique class of cytosolic calcium-dependent proteases that regulate processes as different as cell proliferation, differentiation and migration, apoptosis, and membrane fusion by targeting specific protein substrates^1–8^. Loss-of-function mutations in several calpain genes cause human disease^9–14^. Notably, calpain hyperactivity also plays a pathogenic role in metabolic and degenerative diseases such as type-2 diabetes, Duchenne muscular dystrophy, and Parkinson’s and Alzheimer’s diseases via improper processing of key regulatory proteins^2,8,15–18^. Owing to the involvement of calpain dysregulation in human disease, intensive effort has been focused on the identification of mechanisms that regulate calpain activity. A well-studied modulator of calpain activity is cytosolic calcium^19,20^. The levels of calcium required to maximally activate calpains, however, do not exist within living cells (except under certain pathological contexts)^2,6,21^, indicating that other pathways regulate calpain activity in normal conditions. In fact, autolysis, phosphorylation, and binding of phospholipids have also been proposed to regulate calpains^2,22^. In addition, while calpains are primarily cytosolic, their dynamic association with the cytoskeleton, cell membrane, components of the secretory pathway, and nuclear membrane suggests tight spatiotemporal regulation^23–25^.

KCTD7 (potassium channel tetramerization domain containing 7) is a protein with unknown function that is defective in progressive myoclonic epilepsy-3 (EPM3), neuronal ceroid lipofuscinosis 14 (CLN14), and opsoclonus-myoclonus syndrome (OMS)^26–28^. Pathogenic *KCTD7* mutations cause epilepsy, progressive ataxia, and severe neurocognitive deterioration^27–29^. The molecular function of KCTD7 has remained uncharacterized, as has the pathogenic mechanism linking KCTD7 loss of function to disease^30,31^.

KCTD7 has been shown to interact with the Cullin-3 ubiquitin ligase complex^27,32,33^. Other members of the KCTD protein family have also been shown to interact with cullin-ring-ligases (CRLs) and function as adapters for certain substrates^34–39^. The interaction between KCTD and cullin proteins is mediated by the Bric-a-brack, Tram-track, Broad complex (BTB) domain, a relatively conserved N-terminal domain of KCTD proteins which facilitates homo- or heterodimerization and protein–protein interaction^39–41^.

By using proteomics and cell biology approaches, here we show that the KCTD7–Cullin-3 ubiquitin ligase regulates calpain activity by non-degradative ubiquitination at specific protein sites. Loss of calpain ubiquitination leads to calpain hyperactivation, aberrant cleavage of downstream targets, and cell death associated with caspase-3 activation. The analysis of *Kctd7* knock-out mice shows that loss of KCTD7 leads to higher calpain activity and results in neurodegeneration and behavioral impairment, which are prevented by calpain inhibition. Molecular analyses also show that KCTD7-mediated non-degradative ubiquitination is a common feature shared by the calpain protein family. These results identify an unanticipated regulatory mechanism of calpains and clarify the molecular pathogenesis of loss of KCTD7 function as mediated by calpain hyperactivity.

## Results

### KCTD7 is a component of the Cullin-3 E3 ligase complex and interacts with calpains

**Fig. 1:**
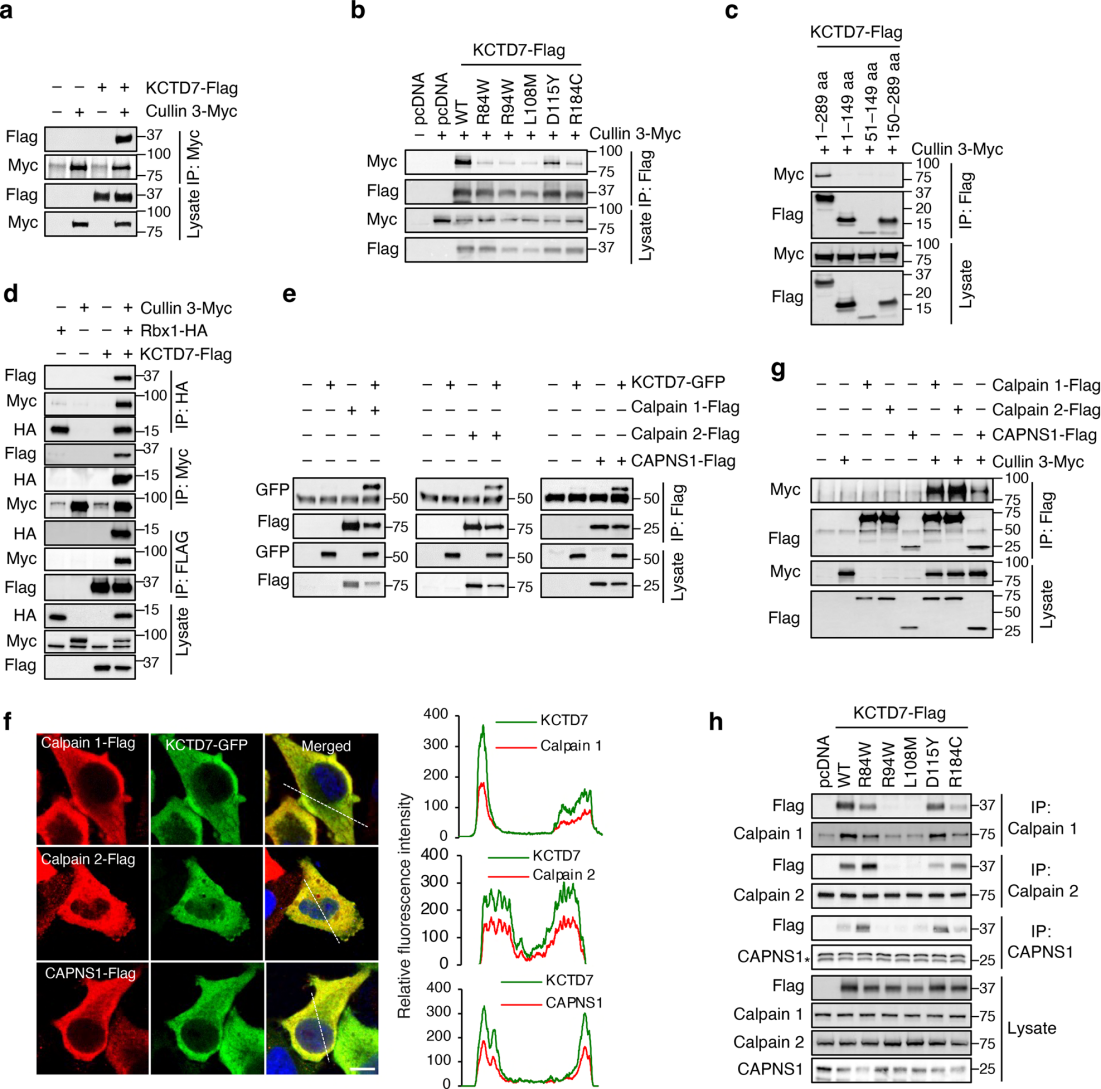
KCTD7 is a component of the Cullin-3 E3 ligase complex and interacts with calpains. **a** Co-IP analysis of Flag-tagged KCTD7 and myc-tagged Cullin-3 in HEK293 cells. **b** Co-IP analysis of KCTD7 mutant constructs and Cullin-3-Myc in HEK293 cells. **c** Co-IP analysis of KCTD7 deletion constructs and Cullin-3-Myc in HEK293 cells. **d** Co-IP analyses of Flag-tagged KCTD7, myc-tagged Cullin-3, and HA-tagged Rbx1 in HEK293 cells. **e** Co-IP analyses of GFP-tagged KCTD7 and Flag-tagged calpain 1, calpain 2, and CAPNS1 in HEK293 cells. **f** Confocal microscopy of HeLa cells showing KCTD7-GFP co-distribute with calpain 1-Flag, calpain 2-Flag, and CAPNS1-Flag. Trace outline is used for line-scan (white dashed line) analysis of relative fluorescence intensity of indicated protein signals. Scale bar, 10 μm. **g** Co-IP analyses of myc-tagged Cullin-3 with Flag-tagged calpain 1, calpain 2 and CAPNS1 in HEK293 cells. **h** Co-IP analyses of Flag-tagged KCTD7 mutant constructs with endogenous calpain 1, calpain 2, and CAPNS1 in HEK293 cells.

To identify candidate KCTD7 targets, we performed tandem affinity purification (TAP) followed by liquid chromatography-tandem mass spectrometry (LC-MS/MS) in HEK293 cell line stably expressing human KCTD7 fused to streptavidin- and calmodulin-binding peptides separated by an HA tag (SBP-HA-CBP-KCTD7) (Supplementary Fig. S1a–d). LC-MS/MS analysis of purified proteins showed that KCTD7 interacts with Cullin-3 as previously reported^27,32^ and also identified several additional interactors (Supplementary Table S1). Immunoprecipitation (IP) of Cullin-3-myc followed by immunoblotting of KCTD7-Flag confirmed the interaction between the two proteins, which was significantly decreased by the introduction of KCTD7 disease-causing mutations (Fig. 1a, b). We also tested KCTD7 deletion constructs for their ability to interact with full-length Cullin-3. The results showed that truncated KCTD7 constructs (N-terminal: amino acids 1–149, BTB-domain only: amino acids 51–149, or C-terminal end: amino acids 150–289) were unable to bind to Cullin-3, indicating that full-length KCTD7 is required for this interaction (Fig. 1c). Cullin-3-associated RING-H2 finger protein Rbx1 (RING Box Protein-1) is the recruiter of ubiquitin-conjugating enzymes (E2s) that catalyzes the transfer of ubiquitin onto substrates^42–45^. Co-immunoprecipitation (Co-IP) experiments confirmed the formation of a Cullin-3–Rbx1–KCTD7 complex (Fig. 1d). Among the proteins that co-purified with KCTD7 were CAPN2 (calpain 2) and CAPNS1, the catalytic and regulatory subunits of m-calpain, respectively. CAPNS1 is also part of μ-calpain, a heterodimer of CAPN1 (calpain 1) and CAPNS1^2^. CAPNS1 is required for the stability of both calpain catalytic subunits in vivo, and probably functions as an intramolecular chaperone^46,47^. Reciprocal co-IP experiments confirmed the interaction between KCTD7 and calpain subunits (Fig. 1e). To identify the protein domains that mediate calpain–KCTD7 interaction, we first performed co-IP experiments using full-length calpain 1 with KCTD7 constructs encoding the full-length protein, its N-terminal domain, or its C-terminal domain. The results showed that full-length KCTD7 is required for its interaction with calpain 1 (Supplementary Fig. S1e). We then co-expressed full-length KCTD7 with constructs carrying combinations of the four calpain 1 domains (domain I to IV) and performed co-IP. The results showed that domain III of calpain 1 is necessary and sufficient for calpain 1 interaction with KCTD7 (Supplementary Fig. S1f). Confocal microscopic analysis of HeLa cells that co-express KCTD7 with either calpain subunits showed that both proteins broadly co-distribute with KCTD7 in the cytosol (Fig. 1f). Similarly, subcellular fractionation of HeLa cells by differential velocity centrifugation showed that endogenous KCTD7 and calpain subunits are all highly enriched in the cytosol (Supplementary Fig. S1g).

Co-IP also revealed interactions between Cullin-3 and calpain subunits (Fig. 1g). Calpain–KCTD7 interactions were hindered by certain disease-associated KCTD7 point mutations (Fig. 1h). Finally, knock-down of calpain subunits did not affect KCTD7–Cullin-3 interaction (Supplementary Fig. S1h–j). Together, these data indicate that KCTD7 is a component of the Cullin-3 E3 ligase complex, where it functions as an adapter for calpain subunits.

### KCTD7–Cullin-3 ubiquitin ligase regulates calpain activity

**Fig. 2:**
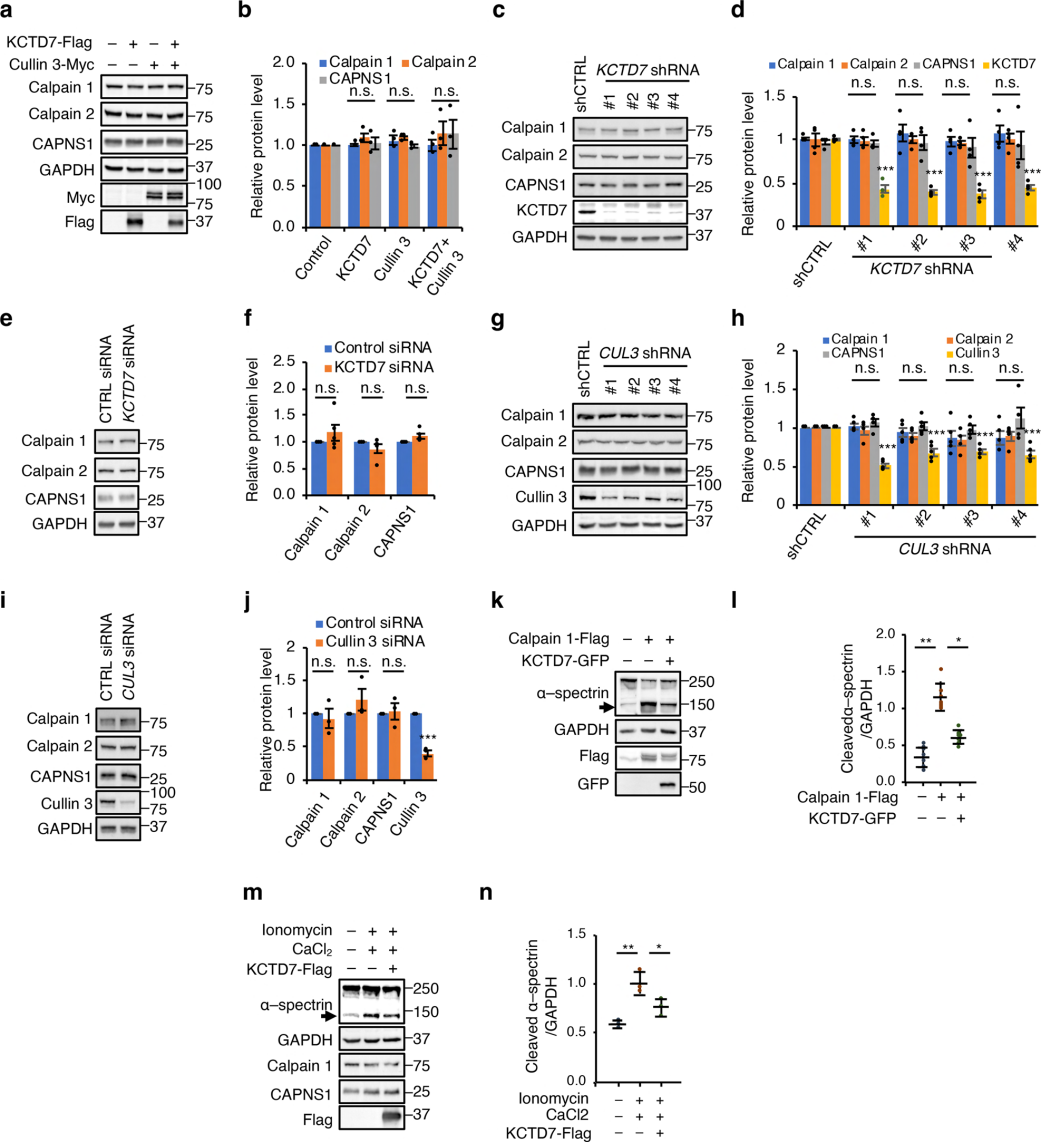
KCTD7–Cullin-3 ubiquitin ligase regulates calpain activity. **a**, **b** Cullin-3 and KCTD7 were expressed in HEK293 cells alone or in combination for 36 h. Lysates were probed with antibodies as indicated, *n* = 4. **c**–**f** KCTD7 was knocked down using shRNAs (**c**, **d**) or siRNA (**e**, **f**) in HEK293 cells for 48 h. Cell lysates were probed with antibodies for calpain subunit and KCTD7 as indicated. GAPDH served as a loading control, *n* = 4. **g–j** Cullin-3 was knocked down by shRNAs (**g**, **h**) or siRNA (**i**, **j**) in HEK293 cells for 48 h. Cell lysates were probed with antibodies for calpain subunit and Cullin-3 as indicated. GAPDH served as a loading control, *n* = 4. **k**, **l** Calpain 1 was expressed alone or in combination with KCTD7 in HEK293 cells and α-spectrin cleavage was evaluated by immunoblotting, *n* = 5. **m**, **n**, HEK293 cells were treated with ionomycin/CaCl_2_ alone or in combination with KCTD7 expression and α-spectrin cleavage was evaluated by immunoblotting, *n* = 3. Data represent means ± SEM; ^∗^*P* < 0.05, ^∗∗^*P* < 0.01, ns not significant.

To test whether calpains are targeted by the KCTD7–Cullin-3 complex for proteasomal degradation, we first checked whether modulation of the levels of the KCTD7–Cullin-3 complex leads to changes in proteins levels of calpain subunits. Transient expression of Cullin-3 and KCTD7 either alone or in combination in HEK293 cells did not lead to any changes in the levels of calpain subunits (Fig. 2a, b), nor did siRNA- or shRNA-mediated knockdown of endogenous *KCTD7* or *CUL3* (Fig. 2c–j and Supplementary Fig. S1k–l). Similarly, CRISPR-mediated knock-out of *KCTD7* in HeLa cells did not result in the accumulation of any calpain subunits (Supplementary Fig. S1m, n).

We therefore sought to determine whether KCTD7–Cullin-3 affects the activity of calpains. Overexpression of calpain 1 resulted in greater rates of cleavage of the cytoskeletal protein α-spectrin, a known substrate of calpain 1 and calpain 2; this increase was reduced by co-expressing KCTD7 (Fig. 2k, l). Expression of KCTD7 also reduced the activity of endogenous calpains induced by ionomycin and CaCl_2_ treatment (Fig. 2m, n and Supplementary Fig. S1o, p). These data indicate that the KCTD7–Cullin-3 complex regulates the activities of calpain 1 and calpain 2 without affecting their protein levels.

### KCTD7 regulates calpain activity via atypical ubiquitination

**Fig. 3:**
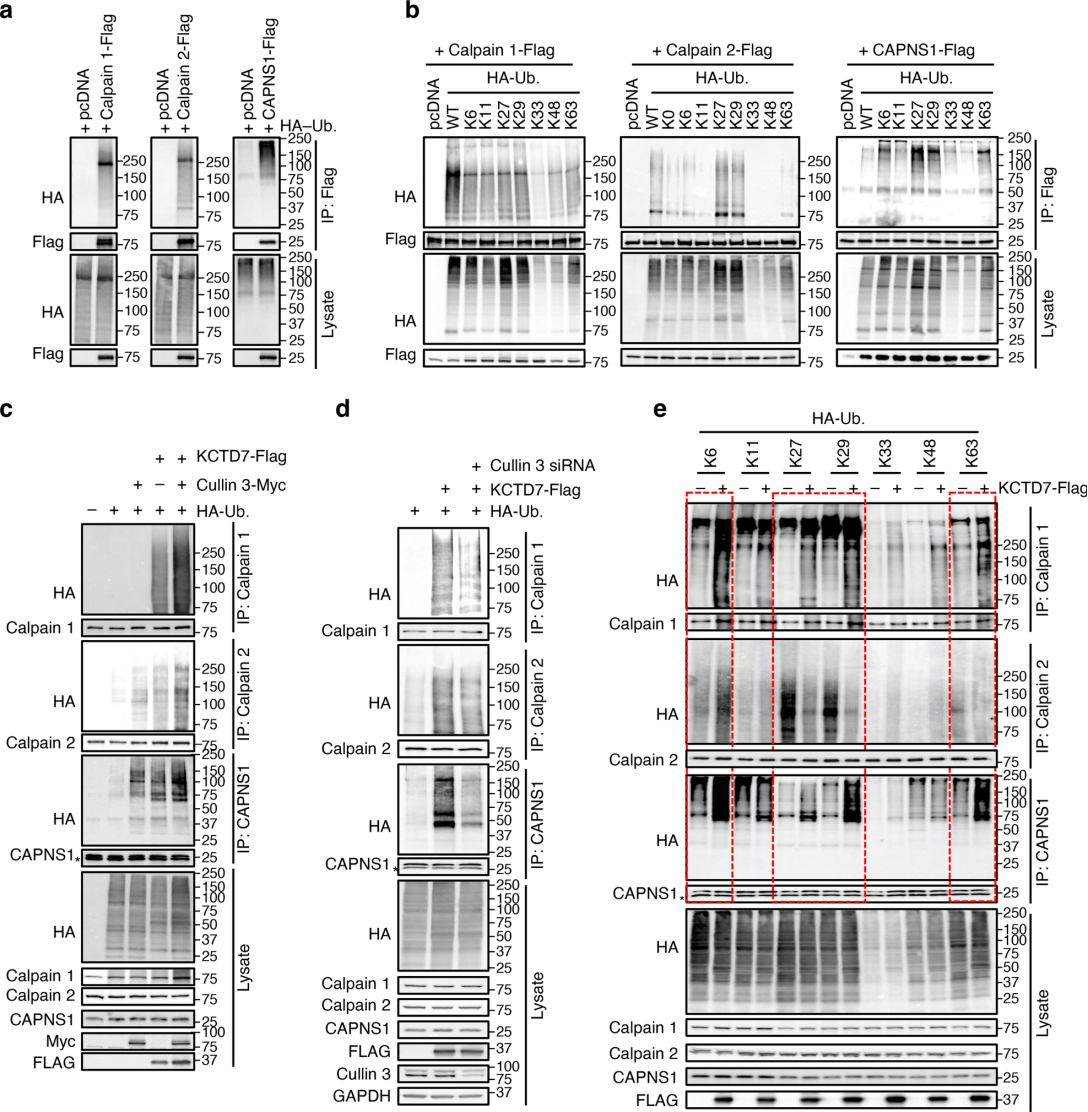
KCTD7–Cullin-3 ubiquitin ligase regulates calpain activity via atypical ubiquitination. **a** In vivo ubiquitination assay for calpain 1, calpain 2, and CAPNS1 performed by coexpressing either protein with HA-Ub in HEK293 cells for 36 h. **b** In vivo ubiquitination assay in HEK293 cells for calpain 1, calpain 2, and CAPNS1 using the indicated ubiquitin mutants. HA-Ub K0 has all lysine residues mutated. **c** In vivo ubiquitination assay for calpain 1, calpain 2, and CAPNS1 performed by expressing Cullin-3 and KCTD7 alone or in combination in HEK293 cells for 36 h. **d** In vivo ubiquitination assay for calpain 1, calpain 2, and CAPNS1 performed upon simultaneous expression of KCTD7 and knock-down of Cullin-3 in HEK293 cells for 48 h. **e** In vivo ubiquitination assay in HEK293 cells for calpain 1, calpain 2, and CAPNS1 performed by expressing the indicated ubiquitin mutants alone or in combination with KCTD7.

An in vivo ubiquitination assay showed that calpain 1, calpain 2, and CAPNS1 all undergo ubiquitination (Fig. 3a). Inhibition of proteasomal degradation by MG132, however, did not lead to the accumulation of any of them (Supplementary Fig. S2a–d) nor resulted in their increased ubiquitination (Supplementary Fig. S2e–g), thereby suggesting that these ubiquitination events are non-proteolytic in nature.

We next investigated which type of ubiquitin-chain linkage is preferred for modification of calpain 1, calpain 2, and CAPNS1. The ubiquitin molecule contains seven lysine residues (K6, K11, K27, K29, K33, K48, and K63), any of which could mediate ubiquitin chain elongation (polyubiquitination). We executed an in vivo ubiquitination assay by expressing calpain subunits with either wild-type HA-ubiquitin or ubiquitin mutants in which all but one of their seven lysine residues were substituted with arginine. The results showed that calpain 1 is modified using most of the ubiquitin lysine residues, calpain 2 is preferably modified via K27 and K29, and CAPNS1 is preferably modified via K6, K27, K29, and K63 (Fig. 3b). A time-course analysis upon treatment with the calpain inducers, ionomycin and CaCl_2_, showed differential ubiquitination dynamics, indicating activity-dependent changes in ubiquitination of calpain subunits (Supplementary Fig. S2h). Additional testing showed that KCTD7 overexpression dramatically increased ubiquitination of calpain 1, calpain 2, and CAPNS1, and co-expression of Cullin-3 further increased ubiquitination of these substrates (Fig. 3c). In vitro ubiquitination assays confirmed that the Cullin-3–Rbx1–KCTD7 complex mediates ubiquitination of calpain subunits (Supplementary Fig. S2i).

**Fig. 4:**
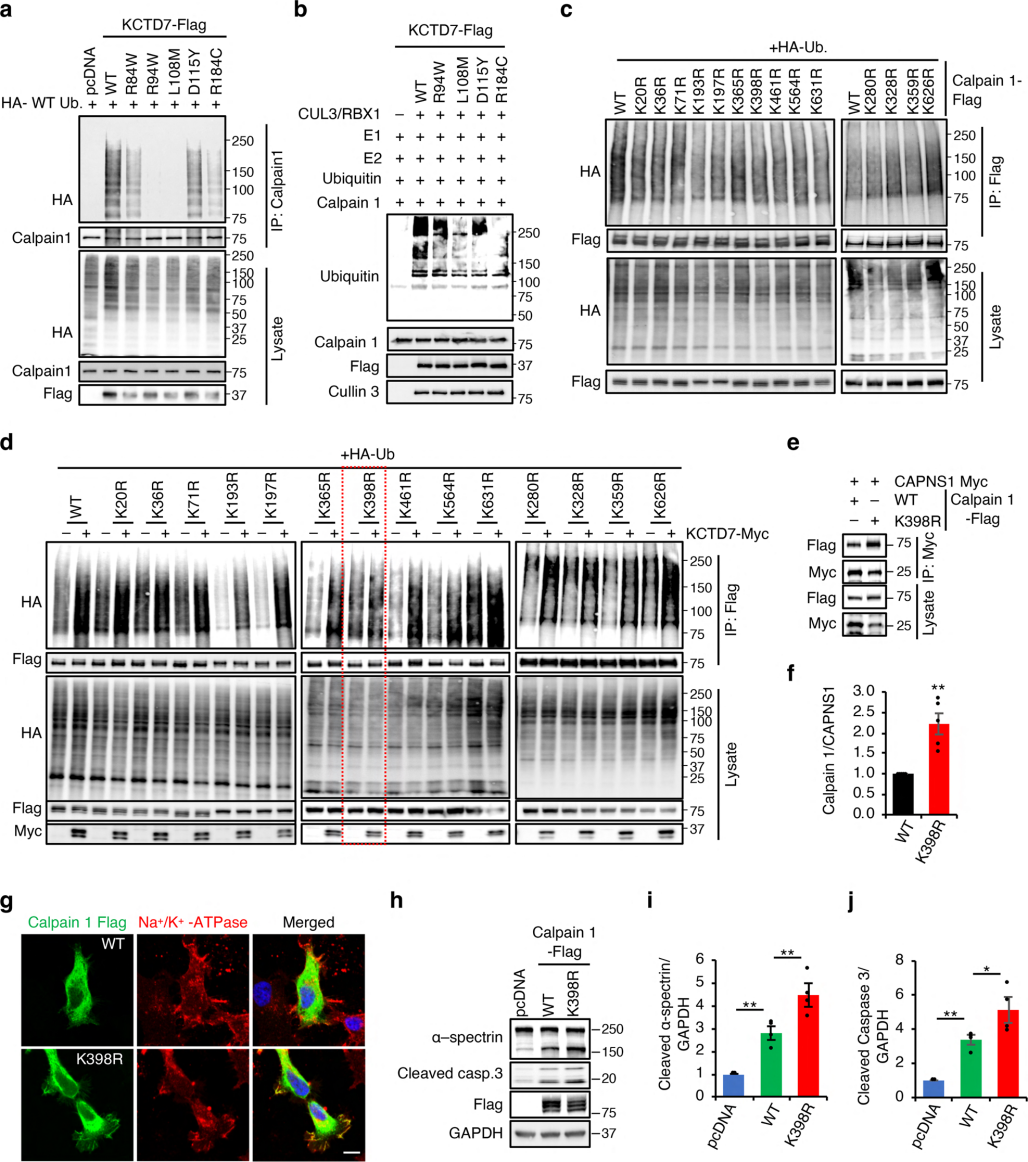
Calpain mutant resistant to KCTD7-mediated ubiquitination is hyperactive. **a** In vivo ubiquitination assay for calpain1 performed by expressing WT KCTD7 or pathogenic mutants in HEK293 cells for 36 h. **b** In vitro ubiquitination assay for calpain 1. **c** In vivo ubiquitination assay in HEK293 cells for calpain 1 K–R mutants. **d** In vivo ubiquitination assay for calpain 1 K–R mutants performed with or without KCTD7 in HEK293 cells. **e**, **f** Co-IP analysis of Myc-tagged CAPNS1 and Flag-tagged WT calpain 1 or calpain 1-K398R in calpain 1 knock-out HEK293 cells, *n* = 5. **g** Confocal microscopy of HeLa cells showing distribution of WT calpain 1 or calpain 1-K398R. Na^+^/K^+^-ATPase was used as plasma membrane marker. Scale bar, 10 μm. **h–j** WT calpain 1 or K398R mutant were expressed in calpain 1 knock out Hela cells and cleavage of α-spectrin cleavage and caspase 3 were evaluated by immunoblotting, *n* = 5. Data represent means ± SEM; ^∗^*P* < 0.05, ^∗∗^*P* < 0.01, ns not significant.

Knock-down of Cullin-3 reduced KCTD7-mediated ubiquitination of calpain 1, calpain 2, and CAPNS1, demonstrating that Cullin-3 is required for ubiquitination of these proteins (Fig. 3d). Using the ubiquitin mutants, we showed that KCTD7 mediates ubiquitination of calpain 1 and CAPNS1 via the same lysine residues established above (K6, K27, K29 and K63; Fig. 3e), whereas it uses K6-mediated ubiquitination to modify calpain 2 (Fig. 3e). Finally, several disease-causing mutants of KCTD7 were unable to ubiquitinate calpain 1 (Fig. 4a, b).

Together, these data establish that KCTD7–Cullin-3 ubiquitin ligase regulates calpain activity via atypical ubiquitination using distinct polyubiquitination chains.

### KCTD7–Cullin-3 ubiquitinates calpain 1 at K398 and calpain 2 at K280 and K674

Protein sequence analysis of calpains conducted with UbiNet^48^ identified several potential ubiquitination sites (Supplementary Table S2). Using site-directed mutagenesis, we generated several calpain 1 lysine-to-arginine mutants. In vivo ubiquitination assays showed that the highly conserved lysine at position 398 (Supplementary Fig. S2j) is required for KCTD7-mediated ubiquitination of calpain 1 (Fig. 4c, d), which was confirmed by mass spectrometry (Supplementary Fig. S2k, l). Interestingly, K398 resides in protein domain III, which mediates interaction with KCTD7 as shown above. To identify the functional consequences of calpain 1 K398R mutation, we expressed either wild-type (WT) or K398R calpain 1 in *CAPN1* knock-out cells that we generated by using CRISPR/Cas9-mediated genome editing (Supplementary Fig. S2m). To check whether calpain 1 ubiquitination alters its calcium sensitivity, we performed in vitro assays by incubating immunopurified WT and ubiquitination-deficient (K398R) calpain 1 for 1 h at different calcium concentrations. We used calpain autolysis as an indicator of calpain activation. Calpain 1-K398R showed identical calcium sensitivity to WT calpain 1 (Supplementary Fig. S2n). Co-IP with CAPNS1 showed increased interaction of calpain 1-K398R compared to WT calpain 1 (Fig. 4e, f). Microscopic analysis of transiently transfected HeLa cells showed partial mislocalization of calpain 1-K398R to the plasma membrane (Fig. 4g). Immunoblotting analysis showed significantly higher α-spectrin cleavage upon calpain 1-K398R expression compared to the WT construct, indicating calpain hyperactivity (Fig. 4h, i). Cleaved caspase-3, a marker of apoptosis and an indicator of increased calpain activity^49–51^, was also increased upon expression of the calpain 1-K398R mutant compared to the WT construct (Fig. 4h, j). Together, these data demonstrate that Cullin-3–KCTD7 ubiquitin ligase controls calpain 1 activity through ubiquitination at K398 and that loss of ubiquitination affects calpain localization and increases the stability of calpain 1-CAPNS1 complex, thereby resulting in higher calpain activity. Additional mutagenesis experiments identified K280 and K674 as the sites of Cullin-3–KCTD7-mediated ubiquitination of calpain 2 (Supplementary Fig. S3a, b). Of note, neither site corresponds to K398 of calpain 1 (Supplementary Fig. S3c). Thus, KCTD7–Cullin-3 controls calpain 1 and calpain 2 using distinct polyubiquitination chains at different protein sites.

### KCTD7-mediated ubiquitination is a general feature of the calpain protein family

**Fig. 5:**
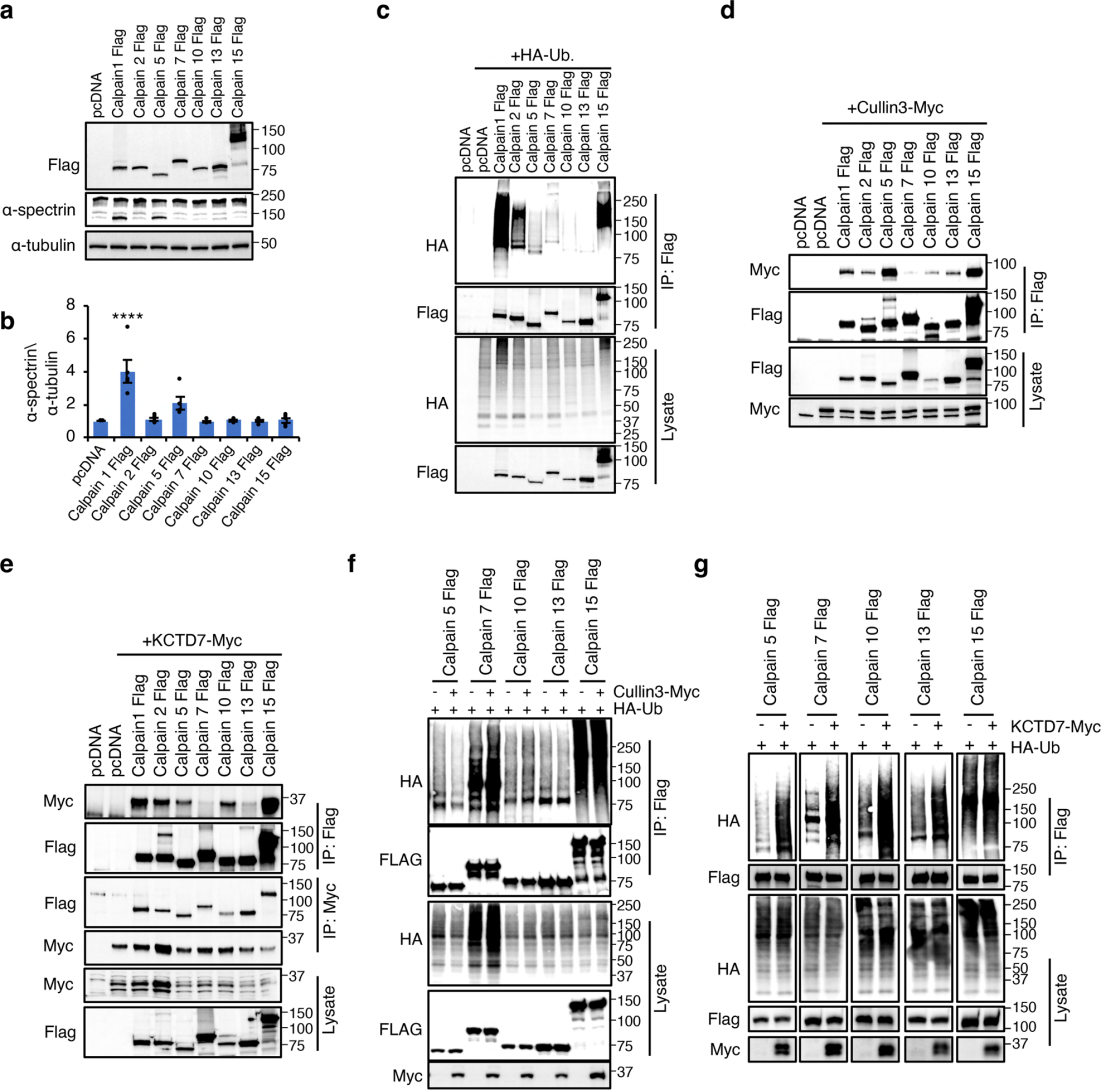
Ubiquitination-mediated regulation is common in calpain family. **a**, **b** Ubiquitous expressing calpains were expressed in HEK293 cells and α-spectrin cleavage was evaluated by immunoblotting, *n* = 3. **c** In vivo ubiquitination assay for calpains performed by coexpressing with HA-Ub in HEK293 cells for 36 h. **d** Co-IP analysis of Flag-tagged calpains and myc-tagged Cullin-3 in HEK293 cells. **e** Co-IP analysis of Flag-tagged calpains and myc-tagged KCTD7 in HEK293 cells. **f** In vivo ubiquitination assay in HEK293 cells for calpains performed by expressing alone or in combination with Cullin-3. **g** In vivo ubiquitination assay in HEK293 cells for calpains performed by expressing alone or in combination with KCTD7.

Next, we investigated whether Cullin-3–KCTD7 ubiquitination is a common feature of the calpain protein family and focused on the ubiquitously expressed family members^9–11,14,52–56^. Exogenous expression of all ubiquitous calpains showed changes in α-spectrin cleavage for a subset of calpains only, suggesting substrate specificity (Fig. 5a, b). In vivo ubiquitination assay showed that all calpains undergo ubiquitination (Fig. 5c). Similar to calpain 1 and 2, proteasomal inhibition by MG-132 or lactacystin did not lead to the accumulation of any of the calpains, indicating that their ubiquitination is not a degradation signal (Supplementary Fig. 4a–h). Co-IP assays showed that all calpains interact with Cullin-3 (Fig. 5d) and KCTD7 (Fig. 5e). Overexpression of KCTD7, but not of Cullin-3, increased the ubiquitination levels of ubiquitous calpains (Fig. 5f, g). Together, these data establish Cullin-3–KCTD7 ubiquitin ligase as a general modifier of the calpain protein family and indicate that the levels of the adapter KCTD7 are a rate-limiting factor for the regulation of calpain activities.

### KCTD7 regulates calpain activity in vivo

**Fig. 6:**
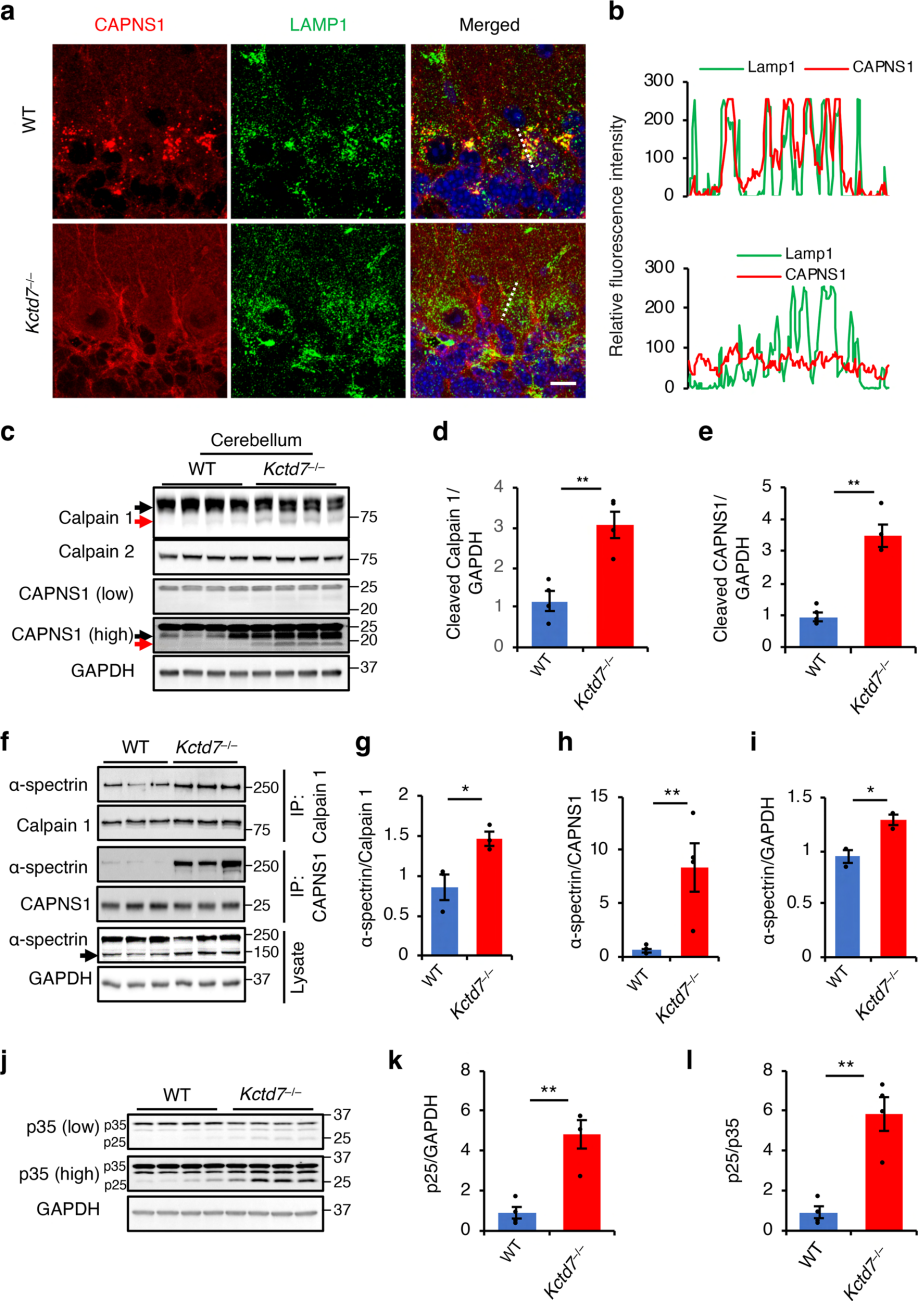
KCTD7 regulates calpain activity in vivo. **a**, **b** Confocal microscopy analysis of cerebellar tissue from 5-week-old WT and *Kctd7*^–/–^ mice showing CAPNS1 co-localization with the lysosomal marker LAMP1 in Purkinje cells. Trace outline is used for line-scan (white dashed line) analysis of relative fluorescence intensity of CAPNS1 and LAMP1 signals. Scale bar, 20 μm. **c**–**e** Cerebellar tissues from 5-week-old WT and *Kctd7*^–/–^ mice were lysed in RIPA buffer and immunoblotted with the indicated antibodies, *n* = 4. **f–l**, Cerebellar tissues from 5-week-old WT and *Kctd7*^–/–^ mice were lysed in NP-40 lysis buffer and calpain 1 and CAPNS1 were immunoprecipitated and probed for interaction with α-spectrin by immunoblotting (**f–h**). Lysates from same tissue samples were also immunoblotted for α-spectrin (**f–i**) and p35 (**j–l**) to evaluate calpain-mediated cleavage, *n* = 4. Data represent means ± SEM; ^∗^*P* < 0.05, ^∗∗^*P* < 0.01, ns not significant.

To study the regulatory role of KCTD7 in vivo, we generated a *Kctd7* knock-out (*Kctd7* KO) mouse line (*Kctd7*^–/–^) by deleting exon 2 using CRISPR/Cas9 genome editing (Supplementary Fig. S5a–d). *Kctd7*^–/–^ mice were viable and fertile and did not show any gross abnormalities, but they had a median lifespan of only eight months (Supplementary Fig. S5e). Immunohistochemical analysis of brain tissue showed that, in WT mice, CAPNS1 displayed a dual subcellular localization in the cytosol and at lysosomes. In *Kctd7*^–/–^ mice, CAPNS1 lost its lysosomal localization and relocated to the plasma membrane (Fig. 6a, b and Supplementary Fig. S5f, h), which is a known indicator of increased calpain activity^23–25^. Immunoblot analysis of brain samples from *Kctd7*^–/–^ mice showed a significantly higher autolysis of calpain 1 and CAPNS1 compared to WT mice (Fig. 6c–e and Supplementary Fig. S5g), another indicator of increased calpain activity^57–59^. To confirm increased calpain activity, we analyzed the interaction of calpains with their substrates and their processing status. Co-IP analyses showed that loss of KCTD7 led to a dramatic increase in the interaction of the ubiquitous calpain substrate, the cytoskeletal protein α-spectrin, with both calpain 1 and CAPNS1 (Fig. 6f–h); α-spectrin cleavage was also markedly increased (Fig. 6f, i). It should be noticed that this increased activity is associated with an overall small fraction of total calpain 1 and CAPNS1 proteins undergoing autolysis. Increased cleavage of α-spectrin was also observed in primary *Kctd7*^–/–^ astrocytes (Supplementary Fig. S5i, j). To check whether calpain activity was increased in neurons, we analyzed the status of the neuron-specific calpain substrate p35^60^. Immunoblot analysis showed that p35-to-p25 conversion was significantly higher in multiple brain regions from *Kctd7*^–/–^ mice than in their WT counterparts (Fig. 6j–l and Supplementary Fig. S5k–p), thus confirming increased neuronal activity of calpains in the absence of KCTD7.

As KCTD7 deficiency has been linked to a subtype of neuronal ceroid lipofuscinosis (NCL)^27^, we checked whether other NCL subtypes are characterized by higher calpain activity. An analysis of the cleavage of α-spectrin and p35 in the brain of mouse models of CLN3, CLN6, and CLN8 diseases showed no obvious differences compared to their WT littermates (Supplementary Fig. S6a–l), indicating specific involvement of KCTD7 in the regulation of calpains.

### KCTD7 deficiency leads to motor incoordination and cerebellar degeneration

**Fig. 7:**
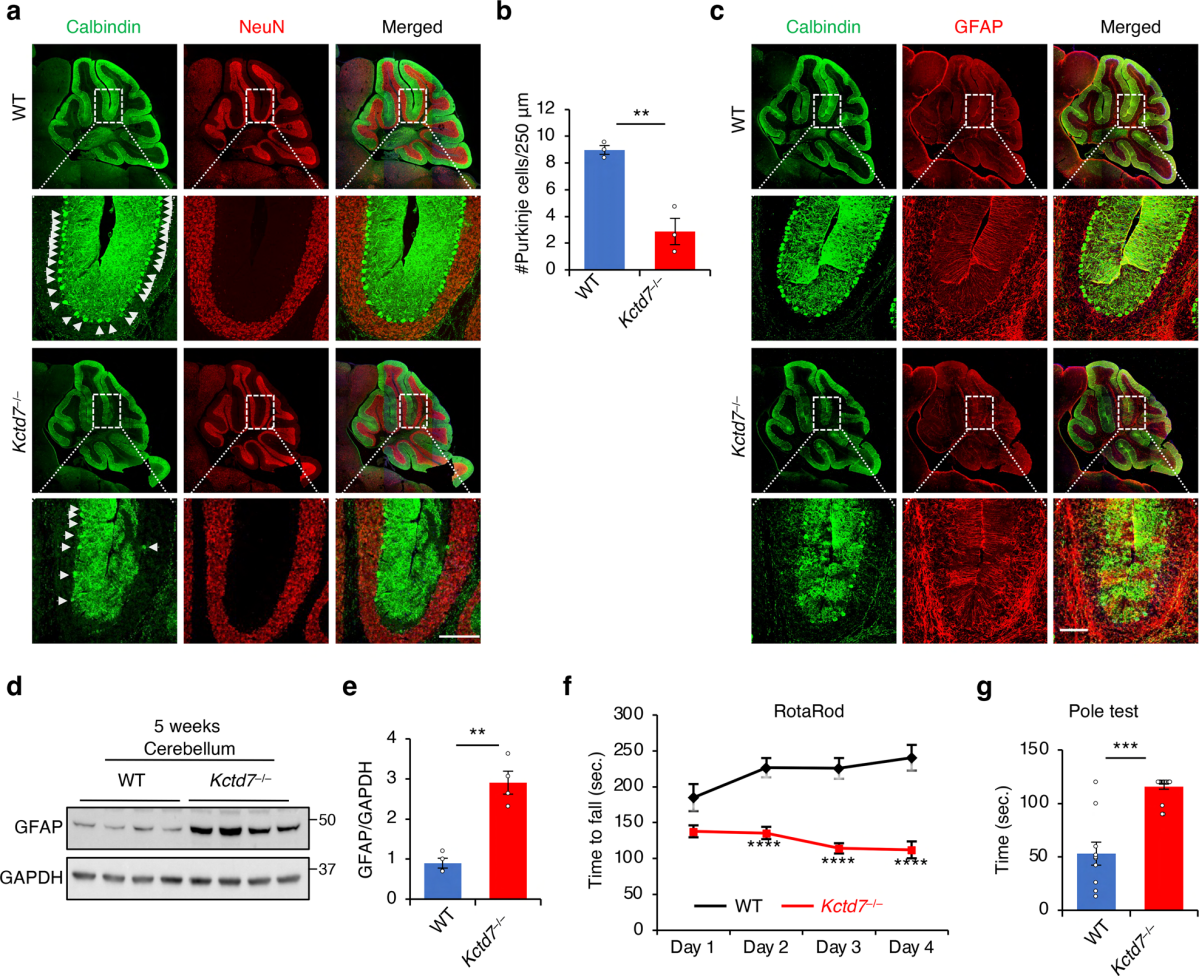
KCTD7 deficiency leads to behavioral impairment and neurodegeneration. **a–c** Confocal microscopy analysis of the cerebellum from 5-week-old WT and *Kctd7*^–/–^ mice. α-calbindin antibody and α-GFAP antibody were used to label Purkinje cells (**a**) and astrocytes (**c**), respectively. Quantification of Purkinje cell numbers is reported in **b**, *n* = 5 per genotype. Scale bars, 200 μm. **d**, **e** Immunoblot analysis of cerebellar tissue from 5-week-old WT and *Kctd7*^–/–^ mice using α-GFAP antibody. **f** Rotarod test measuring the latency to fall for 8-week-old WT (*n* = 10) and *Kctd7*^–/–^ (*n* = 16) mice. **g** Inverted pole test measuring the time used to climb down from the pole for 8-week-old WT (*n* = 10) and *Kctd7*^–/–^ (*n* = 19) mice. Data represent means ± SEM; ^∗^*P* < 0.05, ^∗∗^*P* < 0.01, ^∗∗∗^*P* < 0.001, ns not significant.

Mutations in the *KCTD7* gene cause a spectrum of progressive neurodegenerative phenotypes characterized by ataxia and psychomotor decline/motor incoordination preceded in some (but not all) cases by intractable myoclonic seizures after several months of normal development^26–28^. Progressive decline finally results in severe motor and mental retardation and early death. Additional variable features include dysarthria, truncal ataxia, loss of fine finger movements, and microcephaly^26–28^. Brain imaging shows global cortical and cerebellar atrophy and thinning of the corpus callosum. Differently from other similar progressive neurodegenerative diseases, however, there is an absence of retinal degeneration^27,33,61^. Prior work has shown that *Kctd7* expresses ubiquitously, with significantly higher levels in Purkinje cells of the cerebellum^32^. Thus, to identify pathological phenotypes in *Kctd7*^–/–^ mice, we focused primarily on cerebellar tissue and function. Confocal imaging and immunoblot analysis of cerebellar tissue of *Kctd7*^–/–^ mice at 5 weeks and 6 months of age revealed a severe loss of Purkinje cells associated with caspase-3 activation (an indicator of cell death) at both time points (Fig. 7a, b and Supplementary Fig. S7a–e). Neurodegeneration was accompanied by neuroinflammation, as evidenced by a marked increase in GFAP immunoreactivity (a marker of neuroinflammation) compared to the WT littermates (Fig. 7c–e and Supplementary Fig. S8a, b). In contrast, there was no increase in astrogliosis in the cortex or hippocampus (Supplementary Fig. 8c–f).

We assessed motor performance of *Kctd7*^–/–^ mice at 8 weeks of age using the accelerating rotating rod (rotarod) test and found that the performance of *Kctd7*^–/–^ mice was significantly impaired compared to their WT littermates (Fig. 7f). In a vertical pole test, *Kctd7*^–/–^ mice placed at the top of the pole took significantly longer than their WT littermates to climb down (Fig. 7g). We did not observe any significant differences in muscle strength as measured by the wire suspension test, grid suspension test (Supplementary Fig. S8g, h), or forepaw grip strength test (Supplementary Fig. S8i), confirming that the observed motor incoordination was not due to muscle weakness.

### Calpain inhibition in *Kctd7*^–/–^ mice ameliorates behavioral impairments and neurodegeneration

**Fig. 8:**
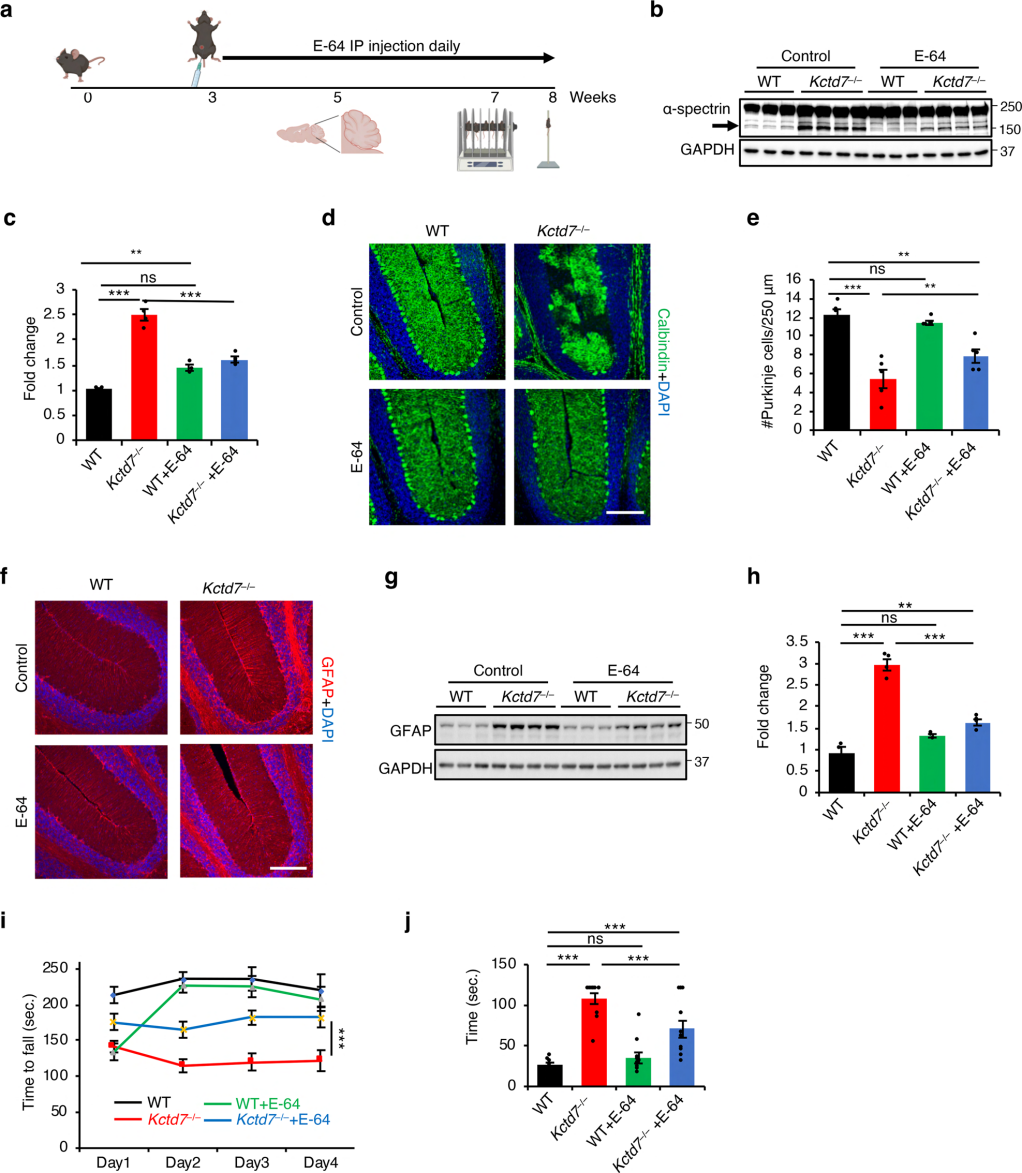
Calpain inhibition in *Kctd7*^–/–^ mice ameliorates behavioral impairments and neurodegeneration. **a** Schematic diagram of drug treatment, tissue preparation and behavioral tests. **b**, **c** Immunoblot analysis of cerebellar tissue from 8-week-old WT and *Kctd7*^–/–^ mice treated with E-64 or vehicle using α-spectrin antibody, *n* = 3 for WT (untreated and treated), *n* = 4 for *Kctd7*^–/–^ (untreated and treated). **d**–**f** Confocal microscopy analysis of the cerebellum from 8-week-old WT and *Kctd7*^–/–^ mice treated with E-64 or vehicle. α-calbindin antibody and α-GFAP antibody were used to label Purkinje cells (**d**) and astrocytes (**f**), respectively. Scale bars, 200 μm. Quantitation of Purkinje cell numbers is reported in **e**, *n* = 5. **g**, **h** Immunoblot analysis of cerebellar tissue from 8-week-old WT and *Kctd7*^–/–^ mice treated with E-64 or vehicle using α-GFAP antibody, *n* = 3 for WT (untreated and treated), *n* = 4 for *Kctd7*^–/–^ (untreated and treated). **i** Rotarod test measuring the latency to fall for 8-week-old WT and *Kctd7*^–/–^ mice treated with E-64 or vehicle. *n* = 10 for vehicle-treated mice (WT and *Kctd7*^–/–^). *n* = 12 for E-64-treated mice (WT and *Kctd7*^–/–^). **j** Inverted pole test measuring the time used to climb down from the pole for 8-week-old WT and *Kctd7*^–/–^ mice treated with E-64 or vehicle. *n* = 10 for vehicle-treated mice (WT and *Kctd7*^–/–^). *n* = 12 for E-64-treated mice (WT and *Kctd7*^–/–^); ^∗^*P* < 0.05, ^∗∗^*P* < 0.01, ^∗∗∗^*P* < 0.001, ns not significant.

To test whether aberrant calpain activity drives neurodegeneration in *Kctd7*^–/–^ mice, we treated them with either the calpain inhibitor E-64 (6.4 mg/kg, i.p. daily) or vehicle for 5 weeks starting at weaning (Fig. 8a)^62^. E-64 is an irreversible, potent, and highly selective cysteine protease inhibitor. The trans-epoxysuccinyl group (active moiety) of E-64 forms an irreversible thiolester bond with the thiol group of the cysteine residue at the active site of the protein^63^. E-64 is suitable for in vivo studies because it permeates tissues and cells and has low toxicity^63^. Calpain inhibition through E64 has been shown to be beneficial in neurodegenerative disease^62,64^. E-64 significantly inhibited calpain activity in vivo as observed by a marked decrease in α-spectrin cleavage (Fig. 8b, c). Neuropathological analyses revealed that calpain inhibition resulted in a significant prevention of Purkinje cell loss (Fig. 8d, e) and in a reduction of astrocytosis (Fig. 8f–h) in *Kctd7*^–/–^ mice. Behavioral testing showed improved performance of *Kctd7*^–/–^ mice upon calpain inhibition in both the rotarod and pole tests compared to vehicle-treated mice (Fig. 8i, j). Together, these data indicate that calpain hyperactivity is a driving factor to neuropathology caused by KCTD7 deficiency.

## Discussion

**Fig. 9:**
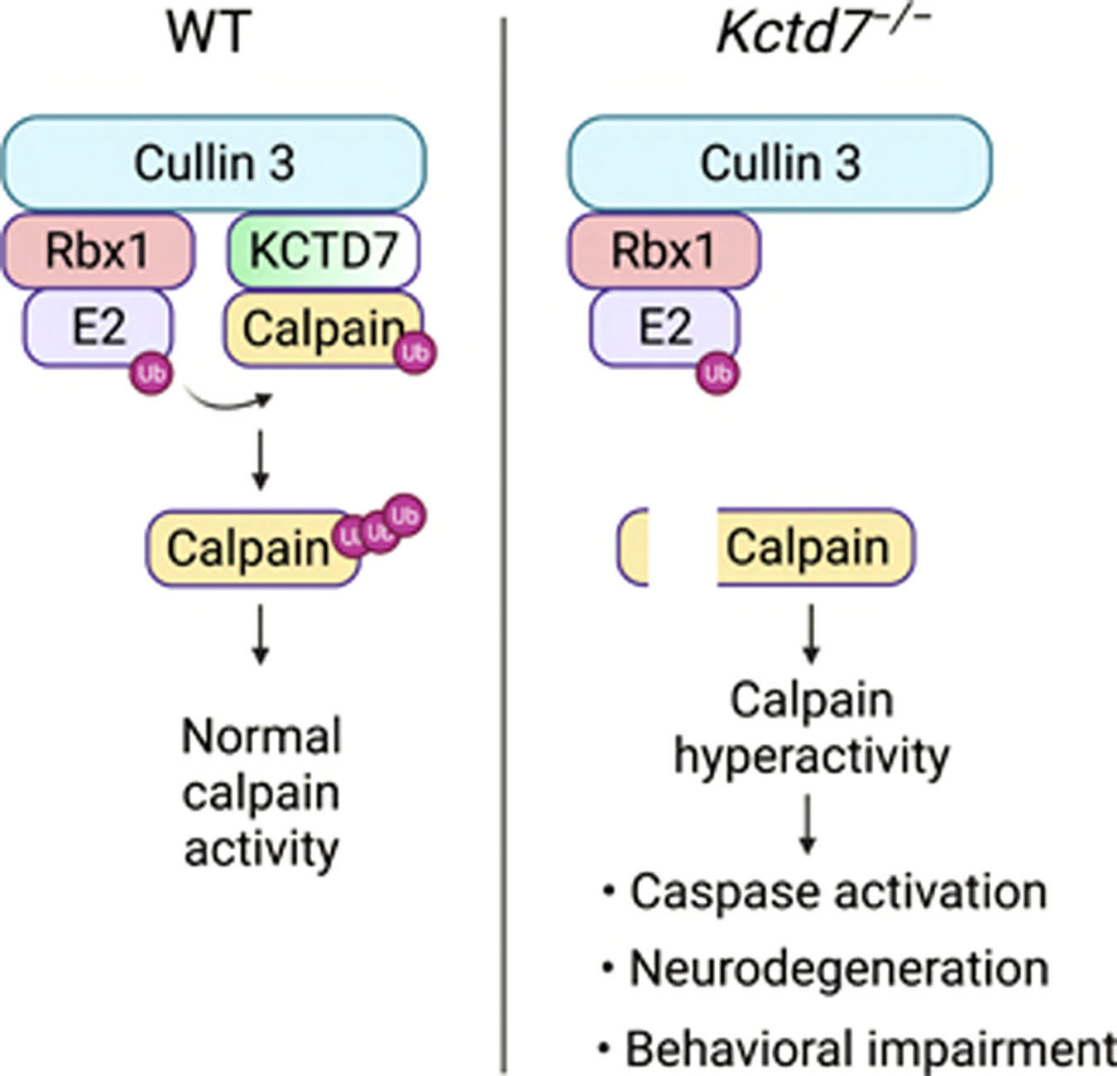
Schematic model of calpain regulation by the Cullin-3–RBX1–KCTD7 complex. Shown is a comparison between WT conditions and deficiency of KCTD7 leading to calpain dysregulation and activation of a pathogenic cascade.

This study establishes that KCTD7 is a key regulator of calpains, a class of non-lysosomal calcium-activated cysteine proteases that exert their regulatory functions via limited proteolysis of their substrates^2^. We found that KCTD7 forms a complex with Cullin-3 to work as a substrate adapter that mediates calpain ubiquitination. Our results show that such ubiquitination is non-degradative and prevents calpain autolysis, thereby resulting in modulation of calpain function (Fig. 9). Interestingly, other members of the KCTD protein family have been shown to interact with Cullin-3, but they target their protein substrates to the ubiquitin-proteasome pathway for degradation^65^. Previous work has shown that several KCTD proteins participate in apoptosis and cell proliferation, differentiation, and metabolism, and loss-of-function mutations in at least 4 KCTD genes cause human disease^29,66–72^. At the molecular level, KCTD proteins have been implicated in the regulation of transcription, DNA replication, amino acid signaling to mTORC1, and regulation of Rho GTPases in brain development among other processes^65^. Thus, there are significant divergences among the mechanisms of action of KCTD proteins as well as the processes they regulate, with KCTD7 being the first example of pathway regulation via non-degradative ubiquitination.

We observed that loss of KCTD7 in mice leads to higher calpain activity in the brain, which is associated with motor impairment, severe loss of Purkinje cells, neuroinflammation, and premature death. Pharmacological inhibition of calpains ameliorated behavioral phenotypes and neuropathology in *Kctd7*^–/–^ mice, thus demonstrating that calpain dysregulation is a key driver in the pathogenesis of disease. Previous work established that KCTD7 regulates retinal neurovascular patterning and function^73^ and also plays a role in the maintenance of autophagic homeostasis^33^ — the latter being a characteristic shared by various NCL proteins^74–78^. Our data, however, show that calpain hyperactivity is a specific consequence of KCTD7 loss of function among several NCL proteins investigated by using mouse models of disease.

Abnormal calpain activity has been shown to contribute to various metabolic and degenerative diseases, especially late-onset neurodegenerative disorders^2,8,15,16,79–81^. Work performed in animal models of frontotemporal dementia, Parkinson’s disease, Huntington’s disease, and spinocerebellar ataxia type 3 has shown that calpain-mediated fragmentation of the proteins implicated in disease (TDP-43, α-synuclein, huntingtin, and ataxin-3) either increases their tendency to aggregate or directly leads to neurotoxicity^82–85^. The emerging consensus is that interventions based on the modulation of calpain activity could be a viable avenue to treat diseases with calpain-associated toxicity; indeed, pharmacological calpain inhibitors are being tested in clinical trials for several human diseases^86,87^. Classical studies have shown that local calcium concentration and subcellular localization are important factors in the regulation of calpain activity^23,24,88,89^. A more recent study has shown that the membrane protein Ttm50, a subunit of the TIM23 complex in the inner mitochondrial membrane, facilitates calpain activation by mediating calpain localization at the Golgi/ER^25,90^. Ttm50 binding increases calpain sensitivity to calcium, possibly either by promoting a calpain conformational change, or by increasing the binding affinity for calcium. Thus, Ttm50 acts as a calpain anchor by localizing calpain to the calcium stores at the Golgi/ER. Similarly, in KCTD7-mediated control of calpain activity, subcellular localization of calpains appears to play an important role in their activation. Ubiquitination-deficient calpain 1-K398R indeed shows altered localization to the plasma membrane. This change does not seem to be coupled with calcium sensitivity but is rather associated with significantly stronger binding to CAPNS1, which is required for calpain 1 stability and activation^90^. Our results uncover a new mechanism of calpain regulation that could be harnessed to restrain abnormal activity of calpains for therapeutic applications by leveraging the cellular components that execute such control.

Using mutants of ubiquitin where only single lysine residues were available for linkage, we determined that the KCTD7–Cullin-3 ligase complex promotes the preferential incorporation of Ub-K6, Ub-K27, Ub-K29, and Ub-K63 for ubiquitination of calpain 1 and CAPNS1, whereas it uses Ub-K6 for calpain 2. Different polyubiquitin chain linkages direct substrates towards different pathways^91^. The functions of these lysine residues are less characterized compared to K48, which instead has a well-defined role in proteasomal-mediated protein degradation^92–94^. Recent work has demonstrated that atypical ubiquitination can regulate protein activity, localization, and affinity to binding partners^95^. Of note, previous work has shown that loss of ubiquitin K6 alters the ubiquitin-proteasome system (UPS) and results in Ca^2+^ elevation, hyperactivation of calpains, and consequent cleavage of calpain substrates^96^. Thus, our finding that ubiquitin K6 is used for ubiquitination-mediated regulation of the calpains provides a mechanistic understanding of these results and links functionally calpain activity to the UPS. We also identified K398 of calpain 1 and K280 and K674 of calpain 2 as the specific sites that undergo Cullin-3–KCTD7-mediated ubiquitination. Our experiments show that ubiquitination-resistant calpain 1-K398R is hyperactive and activates caspase-3, thus demonstrating the role of ubiquitination in the regulation of calpain activity and bridging the link with downstream disease-associated pathways.

In summary, this study establishes a new paradigm for calpain regulation based on KCTD7-mediated atypical ubiquitination. The identification of the function of KCTD7 as a calpain adapter sheds light on the molecular pathogenesis of KCTD7-associated disease and suggests novel therapeutic avenues to mitigate the pathological effects of calpain hyperactivity in neurodegenerative disease and cancer.

## Materials and methods

### Antibodies and reagents

Antibodies used in this study are reported in Supplementary Table S3. Reagents and chemicals include: Ionomycin calcium salt (Sigma # I3909), Protein G-Agarose (Sigma #11243233001), Streptavidin Sepharose High Performance bead (Sigma #17-5113-01), Calmodulin Sepharose 4B (Sigma #17-0529-01), Dynabeads™ Protein G for Immunoprecipitation (Thermo Fisher Scientific #10004D), jetPRIME®, DNA and siRNA transfection reagent, Polyplus-transfection® reagent (VWR #89129-922), TRIzol™ Reagent (Thermo Fisher Scientific #15596018), Quantitect reverse transcriptase (Qiagen #205313), Xpert Protease inhibitor cocktail (GenDEPOT #P3100-100), Xpert Phosphatase inhibitor cocktail (GenDEPOT #P3200-020), Penicillin-Streptomycin (GenDEPOT #CA005-100), 10× PBS Buffer (GenDEPOT #P2100-100), DMEM, High Glucose with _L_-Glutamine (GenDEPOT #CM002-050), Opti-MEM™ Reduced Serum Medium (Thermo Fisher Scientific #31985070), FBS Opti-Gold, Us Origin (GenDEPOT #F0900-050), iTaq Universal SYBRÂ® Green Supermix (Bio-Rad #1725124), Immun-Blot PVDF Membrane (Bio-Rad #1620177), TGX™ FastCast™ Acrylamide Kit (Bio-Rad #161-0173, #161-0175), Precision Plus Protein™ Dual Color Standards (Bio-Rad #1610394), Blotto, non-fat dry milk (Santa-Cruz # sc-2325), SuperSignal™ West Dura Extended Duration Substrate (Thermo Fisher Scientific #34076), VECTASHIELD HardSet Antifade Mounting Medium with DAPI (Vectorlabs #H-1500).

### Plasmid constructs and siRNA

cDNAs generated by retrotranscription of RNAs from HeLa and HEK293 cells using QuantiTect Reverse Transcription kit were used to PCR-amplify the coding sequences of KCTD7 and CAPNS1, which were then inserted into p3×Flag-CMV-14 vector (pcDNA) by using the In-Fusion cloning kit (Clontech). Oligonucleotides used for In-Fusion cloning are reported in Supplementary Table S4. The following plasmids were obtained from AddGene: Tandem affinity purification plasmid pIRESpuro-GLUE (pGLUE) empty backbone (#15100, deposited by Randall Moon); pcDNA3-myc-CUL3 (#19893, deposited by Yue Xiong); p3×Flag-CAPN1 (#60941, deposited by Yi Zhang), p3×Flag-CAPN2 (#60942, deposited by Yi Zhang); Flag-CAPN1 and Flag-CAPN2 (#60941 and #60942, deposited by Yi Zhang), HA-ubiquitin WT, HA-ubiquitin K0, HA-ubiquitin K33, HA-ubiquitin K48, HA-ubiquitin K63 (#17608, #17603, #17607, #17605, and #17606, deposited by Ted Dawson), HA-ubiquitin K6, HA-ubiquitin K11, HA-ubiquitin K27, HA-ubiquitin K29 (#22900, #22901, #22902, and #22903, deposited by Sandra Weller). siRNAs were obtained from Santa-Cruz; KCTD7 (#sc-89656), Cullin-3 (#sc-35130), Calpain 1 (#sc-29885), and Calpain 2 (#sc-41459), Calpain reg. (#sc-29887). ON-TARGETplus Non-targeting Pool of siRNAs was purchased from Dharmacon (#D-001810-10-05). shRNAs targeting Cullin-3 or KCTD7 were obtained from Cell-Based Assay Screening Service (C-BASS) at Baylor College of Medicine.

### Cell culture, lysate preparation, and western blot analysis

HeLa or HEK293 cells (obtained and certified from ATCC) were cultured in DMEM containing 10% FBS and antibiotics (Penicillin/Streptomycin) and were free from mycoplasma contamination. siRNAs or shRNAs were transfected using jetPRIME transfection reagent and incubated for 48–72 h prior to western blot analysis. Plasmids were transfected using jetPRIME transfection reagent and left to express from 30 to 72 h, depending on the downstream application. Unless otherwise mentioned, cells were lysed with RIPA buffer (50 mM Tris-HCl, pH 8.0, 150 mM NaCl, 1 % NP-40, 0.5% sodium deoxycholate, 0.1% SDS) supplemented with protease and phosphatase inhibitors on ice for 30 min with vortexing. Lysates were cleared by centrifugation (13,000 rpm/15 min, 4 °C) followed by protein quantification via BCA assay (Pierce). Sample buffer with reducing agent was added to each lysate followed by 5 min incubation at 95 °C. Samples were spun down and run on Tris-Glycine gel, transferred to a PVDF membrane and blocked for 1 h with blocking buffer (5% w/v, dried skimmed milk in Tris-buffered saline, pH 7.4, and 0.2% Tween 20, TBST) prior to overnight primary antibody incubation. Detection was carried out with SuperSignal™ West Dura Extended Duration Substrate reagent. Images were detected with ImageQuant LAS 4000 (GE Healthcare) and quantified by Fiji analysis software.

### Tandem-affinity purification and mass spectrometry

HEK293 cells (2 × 10^8^ cells) constitutively expressing SBP-HA-CBP-tagged KCTD7 were selected and maintained in DMEM containing 1 µg/mL puromycin. A stable cell line expressing low levels of KCTD7 was used for the tandem-affinity purification procedure. Briefly, the cells were lysed with lysis buffer (10% glycerol, 50 mM HEPES-KOH, pH 8.0, 100 mM KCl, 2 mM EDTA, 0.1% NP-40, 2 mM DTT, 10 mM NaF, 0.25 mM NaOVO_3_, and protease inhibitors). The lysates were cleared by centrifugation at 13,000× *g* for 15 min and then incubated at 4 °C with 100 μL of packed streptavidin resin. The beads were washed 5 times, and protein complexes were then eluted from the streptavidin resin in calmodulin-binding buffer supplemented with 2 mM biotin. The second round of affinity purification was performed with 100 μL of calmodulin resin. After several washes, the protein complexes on beads were directly digested with sequencing-grade trypsin (Sigma). The peptide supernatant solution was removed to a new Eppendorf tube. The beads were further extracted with 100 μL of 1:2 (5% formic acid: acetonitrile) solution with shaking at 37 °C for 15 min. The supernatant was pooled with the previous supernatant. The solutions were Speedvac dried. The digests were resuspended in 0.1% formic acid and 5% acetonitrile solution. The concentration of digest was measured using NanoOrange. The resulting peptide mixture was then analyzed by nano liquid chromatography-tandem mass spectrometry (nanoLC-MS/MS) using data-dependent acquisition on cHiPLC nano liquid chromatography system (Eksigent) and TripleTOF 5600 mass spectrometer (ABSCIEX). Acquired spectra were searched against a FASTA file containing the human NCBI sequences by using the ProteinPilot version 4.5 software.

### Immunofluorescence and Immunohistochemistry

Cells were grown on glass coverslips in 24-well plates and were transiently transfected with appropriate plasmids for 24–36 h. Post transfection, coverslips were washed in PBS and fixed with 4% paraformaldehyde for 15 min. Cells were washed 3 times for 5 min each with PBS and permeabilized with 0.1% TritonX-100 in PBS for 5 min. After permeabilization, cells were blocked with 10% goat serum for 1 h. Primary antibody incubation was carried out overnight at 4 °C. After 3–4 washes with PBS (5 min each), coverslips were incubated with fluorophore-conjugated secondary antibody for 1 h. Coverslips were finally washed 3–5 times with PBS and mounted on glass slides with Vectashield DAPI (Vector Laboratories). Images were acquired through a 63× oil immersion objective Zeiss 880 confocal laser microscope (Zeiss, Oberkochen, Germany). For mouse tissue samples, free floating or slide-mounted sections were permeabilized and blocked in PBS containing 0.3% Triton X-100 and 10% normal goat serum. Samples were incubated with the primary antibody overnight in blocking buffer and were washed three times with PBS the next day prior to adding fluorescent-conjugated secondary antibody (either Alexa 488 or Alexa 568, 1:500 concentration) and incubating for 2 h at room temperature. Tissues were then washed three times (10 min each) and slides were coverslipped using Vectashield DAPI and imaged using a Zeiss 880 confocal laser microscope.

### Immunoprecipitation

Cells were transfected with the desired plasmids as described. 36–48 h later, cells were collected and lysis was performed on ice for 30 min with brief vortexing using lysis buffer (1% NP-40, 150 mM NaCl, 50 mM Tris, pH 8.0, 10% glycerol, 1 mM EDTA and protease inhibitors). Cell debris were removed by centrifugation (13,000 rpm/15 min, 4 °C) and pre-cleared with un-conjugated beads. Primary antibodies (2–5 µg) were added to the lysates and incubated with rotation overnight at 4 °C. The next day, Dynabeads were added to the lysate and incubated for 2 h at 4 °C with rocking. Beads were then washed four times with 500 µL of lysis buffer before being eluted in Laemmli buffer at 95 °C for 10 min. A similar procedure was also followed for coimmunoprecipitation using mouse tissue samples.

### Subcellular fractionation

Subcellular fractionation of HeLa cells was performed as described previously^25^. Briefly, four 15-cm plates of HeLa cells were washed with ice-cold PBS with protease inhibitors. Cells were then homogenized in 5 mL of buffer A (0.3 M sucrose, 1 mM EDTA, 1 mM MgSO4, 10 mM MES-KOH, pH 6.5) containing protease and phosphatase inhibitors by 5 freeze-thaw cycles using liquid nitrogen and a 37 °C waterbath. The cell homogenates were centrifuged at 1000× *g* for 10 min to remove unbroken cells and nuclei. The supernatant was next centrifuged at 8000× *g* and 35,000× *g* for 30 min each, which sediments the mitochondria. A subsequent ultracentrifugation (150,000× *g* for 120 min) resulted in separation of Golgi/ER and cytosol. Equal amounts of proteins for each fraction were processed for western blotting analysis.

### In vitro ubiquitination assay

Flag-tagged calpain subunits and KCTD7 protein were immunopurified from transiently transfected HEK293 cells using Flag antibodies and peptides. Purified proteins were incubated with 12 μg of ubiquitin (R&D Systems; U-100H), 120 ng UBE1 (R&D Systems; E-304-050), 300 ng UbcH5b/UBE2D2 (R&D Systems; E2-622-100), 150 ng His6-CUL3/NEDD8/RBX1 Complex Protein (R&D Systems; E3-436-025), and 10 mM MgATP Solution (R&D Systems; B-20) in E3 Ligase Reaction Buffer (R&D Systems; B-71) for 1 h at 37 °C with gentle shaking. The reactions were quenched with addition of the SDS-PAGE sample buffer and boiling and were analyzed by SDS-PAGE.

### Generation of *Kctd7*^–/–^ mice

*Kctd7*^–/–^ mice were generated at Mouse Embryonic Stem Cell Core and Genetically Engineered Mouse Core at Baylor College of Medicine. Two gRNAs were designed to cut both ends of the exon 2 of the *Kctd7* gene (cgacctgatagcccttaaatggg and tgtgtgcgtgaggcccgaatggg). Established methods were used to co-microinject 100 ng/µL Cas9 mRNA and 20 ng/µL validated sgRNA (each) into the cytoplasm of 100 C57BL/6NJ embryos. Following micro-injection, zygotes were transferred into pseudopregnant FVB females. Both C57BL/6 J and FVB mice were purchased from the Jackson Laboratory (Bar Harbor, ME). Potential founder mice were genotyped using tail tissue DNA and deletion was confirmed using Sanger sequencing. Genotyping was done using the primers indicated in Supplementary Table S4. The top ten potential off-target sites for each sgRNA were sequenced to confirm no off-target effects. Founder mice were backcrossed at least three times prior to experimentation to eliminate other potential off-target mutations. All animals were housed in a Level 3, AALAS-certified facility on a 12h-light cycle. Husbandry, housing, euthanasia, and experimental guidelines were reviewed and approved by the Institutional Animal Care and Use Committee (IACUC) of Baylor College of Medicine.

### Rotarod

Eight- to nine-week-old mice were tested. After 30 min habituation in the test room, motor coordination was measured using an accelerating rotating rod (Ugo Basile Biological Research Apparatus, Varese, Italy). Mice were tested for 4 consecutive days, 4 trials each, with an interval of 30 min between trials to rest. Each trial lasted for a maximum of 5 min and the rod accelerated from 4 to 40 rpm. “Latency to fall” was recorded either when the mouse fell from the rod or when the mouse had ridden the rotating rod for two revolutions without regaining control. Behavioral scores were subjected to statistical analysis by two-way ANOVA with Bonferroni’s post-hoc analysis.

### Vertical pole

Animals were habituated to the experimental room for 30–60 min prior to testing. Mice were placed on the top of a rough-surfaced vertical pole (45 cm high, 1.1 cm in diameter). The time for each mouse to turn downward and to reach the floor was recorded. Each trial lasted for a maximum of 120 s and each mouse performed three trials with 5 min inter-trial intervals.

### Grip strength

Mice were habituated in the test room for 30 min. Each mouse was allowed to grab the bar of a digital grip strength meter (Columbus Instruments, Columbus, OH) with both forepaws while being held by the tail and then pulled away from the meter with a constant slow force until the forepaws released. The grip (in kg of force) was recorded, and the procedure repeated twice for a total of three pulls, which were averaged for the final result. Data is shown as means ± SD.

### Wire suspension

The mice were habituated to the experimental room for 30–60 min prior to testing. The mice were forced to grasp a 3‐mm wire and hang from it on their forepaws. The ability of the mice to grasp the wire was scored and the time for which they held the wire (maximum 30 s) was registered.

### Inverted grid

The mice were habituated to the experimental room for 30–60 min prior to testing. A grid screen measuring 20 cm × 25 cm with a mesh density of 9 squares/cm^2^ was elevated 45 cm above a cage with bedding. Each subject was placed head oriented downward in the middle of the grid screen. When it was determined that the subject had proper grip on the screen, it was inverted 180°. The hang time (duration a subject held on to the screen without falling) was recorded, up to a cutoff time of 60 s. Any subject that was able to climb onto the top of the screen was assigned a time of 60 s.

### Mass spectrometry analysis

Trypsin (800 ng) was added to immunoprecipitated proteins on the beads for 6 h at 37 °C. The initial digested samples were centrifuged for 2 min at 5000× *g* and the supernatants were collected into fresh tubes. Beads were washed twice with 100 mM ammonium bicarbonate and the supernatants were pooled. The resulting samples were reduced with 20 mM dithiothreitol at 37 °C for 1 h, and cysteine was alkylated with 80 mM iodoacetamide for 45 min in dark. Samples were treated with 600 ng of trypsin to overnight incubation at 37 °C. The resulting peptides were desalted using solid-phase extraction on a C18 Spin column and eluted with 0.1% FA in 80% ACN. Peptides were analyzed by LC-MS/MS using a nanoElute coupled to a timsTOF Pro2 Mass Spectrometer (Bruker Daltonics). Samples were loaded on a capillary C18 column (15 cm length, 75 μm inner diameter, 1.9 μm particle size, 120 Å pore size; Bruker Daltonics). The flow rate was kept at 300 nL/min. Solvent A was 0.1% FA in water, and Solvent B was 0.1% FA in ACN. The peptides were separated on a 100 min analytical gradient from 2% ACN/0.1% FA to 35% ACN/0.1% FA for a total of 120 min gradient. The timsTOF Pro2 was operated in the PASEF mode. MS and MS/MS spectra were acquired from 100 to 1700 m/z. The inverse reduced ion mobility 1/K_0_ was set to 0.60−1.60 V·s/cm^2^ over a ramp time of 100 ms. Data-dependent acquisition was performed using 10 PASEF MS/MS scans per cycle with a near 100% duty cycle. The resulting protein tandem MS data was queried for protein identification against the SwissProt human database (released on April, 2021) using MaxQuant v2.1.0.0. The following modifications were set as search parameters: peptide mass tolerance at 20 ppm, trypsin digestion cleavage after K or R (except when followed by P), 2 allowed missed cleavage sites, carbamidomethylated cysteine (static modification), and oxidized methionine, deaminated asparagine/glutamine, protein N-term acetylation, and Diglycyl lysine (variable modification). Search results were validated with peptide and protein FDR both at 0.01. High confident ubiquitination sites were verified with high localization probability (> 0.95) and manual confirmation of MS/MS spectrum. The mass spectrometry proteomics data have been deposited to the ProteomeXchange Consortium via the PRIDE partner repository with the dataset identifier PXD038842.

## Supplementary information

Supplementary Information
