# Supplementary material for: Calpain activity is negatively regulated by a KCTD7–Cullin-3 complex via non-degradative ubiquitination
Source: Cell Discov. 2023 Mar 24;9:32. doi: 10.1038/s41421-023-00533-3 (PMC10038992; doi:10.1038/s41421-023-00533-3)
Supplement: Supplementary file 1 — Supplementary Information [file 41421_2023_533_MOESM1_ESM.pdf]

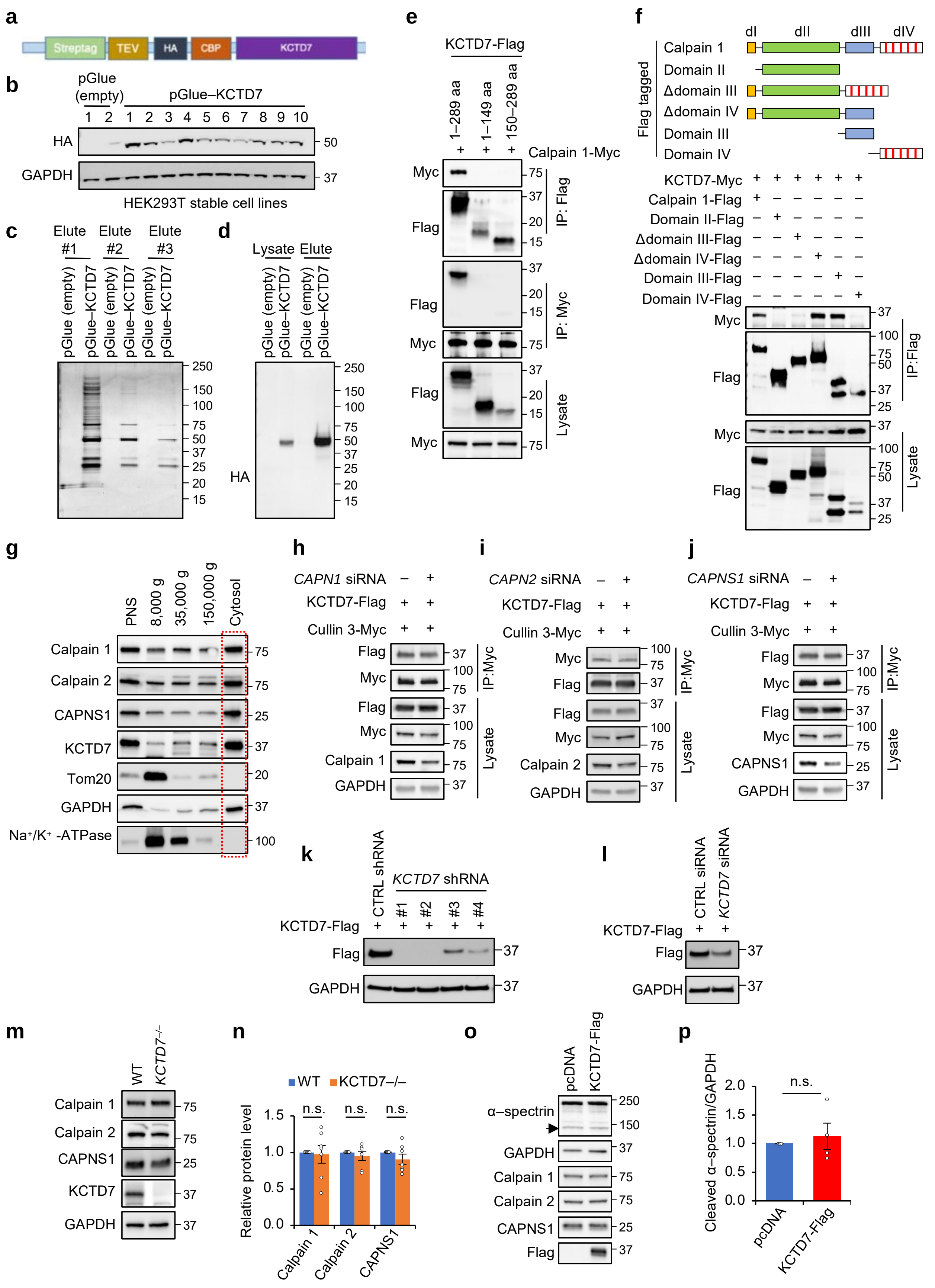
a
Streptag
TEV
HA
CBP
KCTD7
b
pGlue (empty)
pGlue–KCTD7
HA
GAPDH
HEK293T stable cell lines
c
Elute #1
Elute #2
Elute #3
d
Lysate
Elute
e
KCTD7-Flag
1–289 aa
1–149 aa
150–289 aa
Calpain 1-Myc
IP: Flag
IP: Myc
Lysate
f
Flag tagged
Calpain 1
Domain II
Δdomain III
Δdomain IV
Domain III
Domain IV
KCTD7-Myc
Calpain 1-Flag
Domain II-Flag
Δdomain III-Flag
Δdomain IV-Flag
Domain III-Flag
Domain IV-Flag
g
PNS
8,000 g
35,000 g
150,000 g
Cytosol
Calpain 1
Calpain 2
CAPNS1
KCTD7
Tom20
GAPDH
Na+/K+ -ATPase
h
CAPN1 siRNA
KCTD7-Flag
Cullin 3-Myc
i
CAPN2 siRNA
j
CAPNS1 siRNA
k
CTRL shRNA
KCTD7 shRNA
l
CTRL siRNA
KCTD7 siRNA
m
WT
KCTD7–/–
n
WT
KCTD7–/–
n.s.
Relative protein level
o
pcDNA
KCTD7-Flag
α–spectrin
p
Cleaved α–spectrin/GAPDH
n.s.

Fig. S1: Interaction of KCTD7 with Cullin-3 and calpains. a, Schematic representation of the pGLUE-KCTD7 expression vector. Shown are the streptavidin-(streptag) and calmodulin-(CBP) binding affinity tags placed in tandem, along with an HA epitope for protein detection, placed at the N-terminus of full length human KCTD7. **b**, Immunoblot analysis of HEK293 cell clones stably expressing pGLUE-KCTD7 using α-HA antibody. **c**, Silver staining gel analysis of affinity purified proteins. **d**, Immunoblot analysis of affinity purified proteins using α-HA antibody to detect KCTD7. **e,** Co-IP analysis of KCTD7 deletion constructs and calpain 1-Myc in HEK293 cells. **f,** Co-IP analysis of calpain 1 deletion constructs and KCTD7-Myc in HEK293 cells. **g,** Western blot analysis of subcellular fractions of HeLa cell lysates after differential velocity centrifugation. Equal amounts of protein from each fraction were separated by SDS-PAGE. **h-j**, Calpain 1 (h), calpain 2 (i), and CAPNS1 (j) were knocked down in HEK293 cells and, 24 h later, cells were transfected with plasmids expressing KCTD7 and Cullin-3 for 48 h Shown are co-IP analyses of KCTD7 and Cullin-3 in the tested conditions. **k**, **l**, Evaluation of shRNAs and siRNAs for KCTD7. KCTD7-Flag was transfected in HEK293 cells and, 24 h later, cells were transfected with the indicated shRNAs (k) or siRNAs (l) for 48 h. Cell lysates were immunoblotted to detect KCTD7 expression using α-Flag antibody. **m, n**, Immunoblot analysis of *KCTD7* knock-out HeLa cells. **o**, **p**, KCTD7-Flag was expressed in HEK293 cells for 36 h and α-spectrin cleavage was evaluated by immunoblotting using α-spectrin antibody. Data represent mean ± SEM, $n = 3$; $^{*}P < 0.05$, $^{**}P < 0.01$, $^{***}P < 0.0001$, ns = not significant.

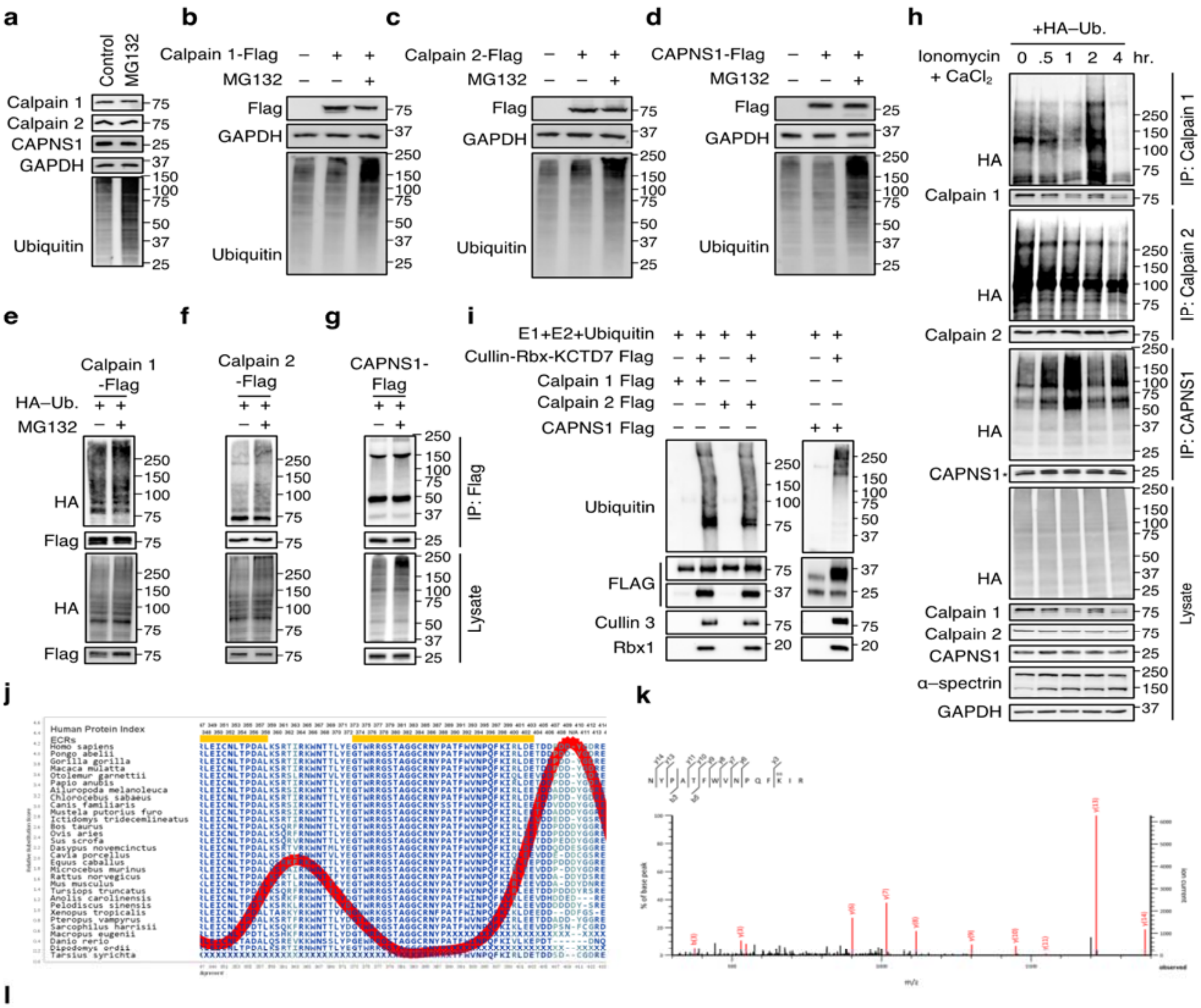

				n=1		n=2	
				pcDNA	KCTD7	pcDNA	KCTD7
Gene name	GlyGly (K) site	GlyGly (K) Probabilities	Sequence window	Intensity	Intensity	Intensity	Intensity
CAPN1	398	NYPATFWVNPQFK(1)IR	GCRNYPATFWVNPQFKIRLDETDDPDDYGDR	0	68607	0	40721

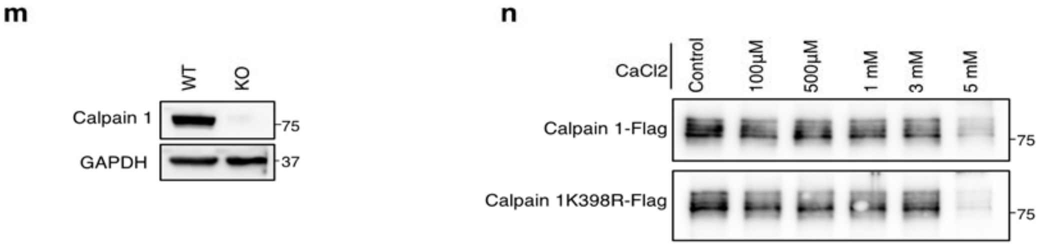

Sup. 2

Fig. S2: Calpains are regulated via ubiquitin-mediated modification. a, Immunoblot analysis of HEK293 cells treated with MG132 (10 μM) or vehicle for 4 h using antibodies for calpain 1, calpain 2, and CAPNS1. GAPDH was used as a loading control. **b-d**, Immunoblot analysis of HEK293 cells expressing calpain 1 (b), calpain 2 (c), or CAPNS1 (d) and treated with MG132 (10 μM) or vehicle for 4 h before protein collection. GAPDH was used as a loading control. **e-g**, In vivo ubiquitination assay for calpain1 (e), calpain 2 (f), and CAPNS1 (g) performed by coexpressing either protein with HA-Ub in HEK293 cells for 36 h and treating with MG132 (10 μM) or vehicle for 4 h before protein collection. **h**, In vivo ubiquitination assay for calpain1, calpain 2, and CAPNS1 performed after treatment with 1 μm Ionomycin along with 5 mM $CaCl_2$ for the indicated times. The lysates were also blotted for α-spectrin to evaluate calpain activity. **i,** In vitro ubiquitination assay of calpain 1, calpain 2, and CAPNS1. **j**, Evolutionarily constrained region analysis of calpain1. Shown is a multi-alignment of calpain1 protein sequences along with a plot of local evolutionary rates. Evolutionarily constrained regions (ECRs) are indicated with yellow lines. **k-l,** Mass spectrometry data of enzymatic digested peptides from CAPN1-Flag was acquired on nanoElute coupled to a timsTOF Pro2 Mass Spectrometer and analyzed using MaxQuant v2.1.0.0. The ubiquitination site of CAPN1-Flag on K398 is highlighted and shown on the peptide sequence. Trypsin digestion of ubiquitin conjugates generates a diGly tag that is formed at the ubiquitinated lysine residue. **m**, Immunoblot analysis of calpain 1 knock out Hela cells. **n**, Immunoblot analysis of calpain autolysis by incubating Flag tagged WT calpain 1 or calpain 1-K398R immunopurified proteins, from HEK293 cells, with indicated calcium concentrations.

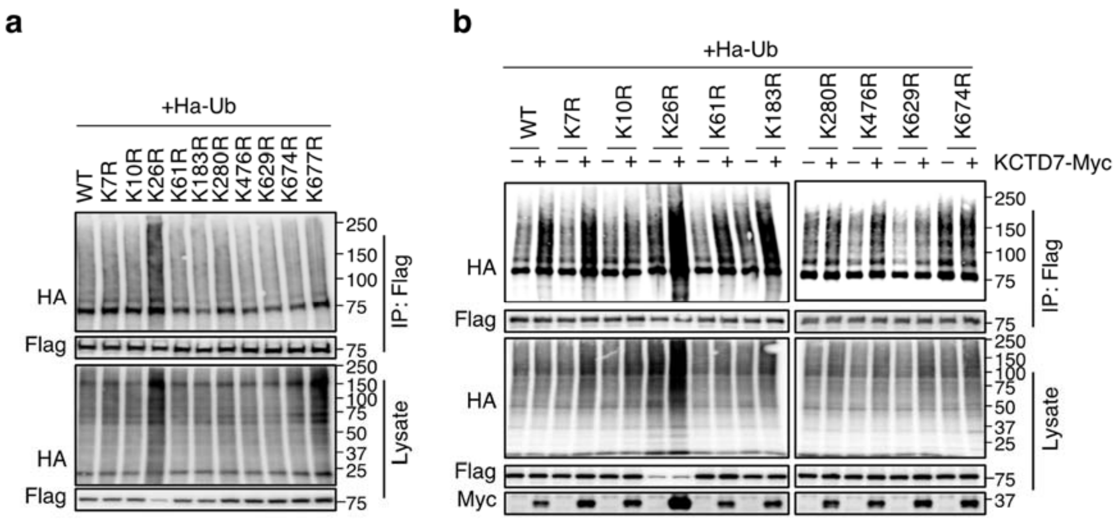

c

```
                1                                                                 60
Calpain 1   MSEEIITPVY CTGVSAQVQK QRARELGLGR HENAIKYLGQ DYEQLRVRCL QSGTLFRDEA
Calpain 2              MAGIAAKLAK DREAAEGLGS HDRAIKYLNQ DYEALRNECL EAGTLFQDPS
            61                                                               120
Calpain 1   FPPVPQSLGY KDLGPNSSKT YGIKWKRPTE LLSNPQFIVD GATRTDICQG ALGDCWLLAA
Calpain 2   FPAIPSALGF KELGPYSSKT RGIEWKRPTE ICADPQFIIG GATRTDICQG ALGDCWLLAA
            121                                                              180
Calpain 1   IASLTLNDTL LHRVVPHGQS FQNGYAGIFH FQLWQFGEWV DVVVDDLLPI KDGKLVFVHS
Calpain 2   IASLTLNEEI LARVVPLNQS FQENYAGIFH FQFWQYGEWV EVVVDDRLPT KDGELLFVHS
            181                                                              240
Calpain 1   AEGNEFWSAL LEKAYAKVNG SYEALSGGST SEGFEDFTGG VTEWYELRKA PSDLYQIILK
Calpain 2   AEGSEFWSAL LEKAYAKING CYEALSGGAT TEGFEDFTGG IAEWYELKKP PPNLFKIIQK
            241                                                              300
Calpain 1   ALERGSLLGC SIDISSVLDM EAITFKKLVK GHAYSVTGAK QVNYRGQVVS LIRMRNPWGE
Calpain 2   ALQKGSLLGC SIDITSAADS EAITFQKLVK GHAYSVTGAE EVESNGSLQK LIRIRNPWGE
            301                                                              360
Calpain 1   VEWTGAWSDS SSEWNNVDPY ERDQLRVKME DGEFWMSFRD FMREFTRLEI CNLTPDALKS
Calpain 2   VEWTGRWNDN CPSWNTIDPE ERERLTRRHE DGEFWMSFSD FLRHYSRLEI CNLTPDTLTS
            361                                                              420
Calpain 1   RTIRKWNTTL YEGTWRRGST AGGCRNYPAT FWVNPQFKIR LDETDDPDDY GDRESGCSFV
Calpain 2   DTYKKWKLTK MDGNWRRGST AGGCRNYPNT FWMNPQYLIK LEEEDEDEE- -DGESGCTFL
            421                                                              480
Calpain 1   LALMQKHRRR ERRFGRDMET IGFAVYEVPP ELVGQPAVHL KRDFFLANAS RARSEQFINL
Calpain 2   VGLIQKHRRR QRKMGEDMHT IGFGIYEVPE ELSGQTNIHL SKNFFLTNRA RERSDTFINL
            481                                                              540
Calpain 1   REVSTRFRLP PGEYVVVPST FEPNKEGDFV LRFFSEKSAG TVELDDQIQA NLPDEQVLSE
Calpain 2   REVLNRFKLP PGEYILVPST FEPNKDGDFC IRVFSEKKAD YQAVDDEIEA NL-EEFDISE
            541                                                              600
Calpain 1   EEIDENFKAL FRQLAGEDME ISVKELRTIL NRIISKHKDL RTKGFSLESC RSMVNLMDRD
Calpain 2   DDIDDGFRRL FAQLAGEDAE ISAFELQTIL RRVLAKRQDI KSDGFSIETC KIMVDMLDSD
            601                                                              660
Calpain 1   GNGKLGLVEF NILWNRIRNY LSIFRKFDLD KSGSMSAYEM RMAIESAGFK LNKKLYELII
Calpain 2   GSGKLGLKEF YILWTKIQKY QKIYREIDVD RSGTMNSYEM RKALEEAGFK MPCQLHQVIV
            661                                                     714
Calpain 1   TRYSEPDLAV DFDNFVCCLV RLETMFRFFK TLDTDLDGVV TFDLFKWLQL TMFA
Calpain 2   ARFADDQLII DFDNFVRCLV RLETLFKIFK QLDPENTGTI ELDLISWLCF SVL
```

Fig. S3: Determination of KCTD7 mediated ubiquitination site in calpain 2. a, In vivo ubiquitination assay in HEK293 cells for calpain 2 K—R mutants. **b**, In vivo ubiquitination assay

for calpain2 K—R mutants performed with or without KCTD7 in HEK293 cells. **c**, Multiple alignment of calpain 1 and calpain 2 proteins. Lysine residues ubiquitinated by KCTD7 are highlighted.

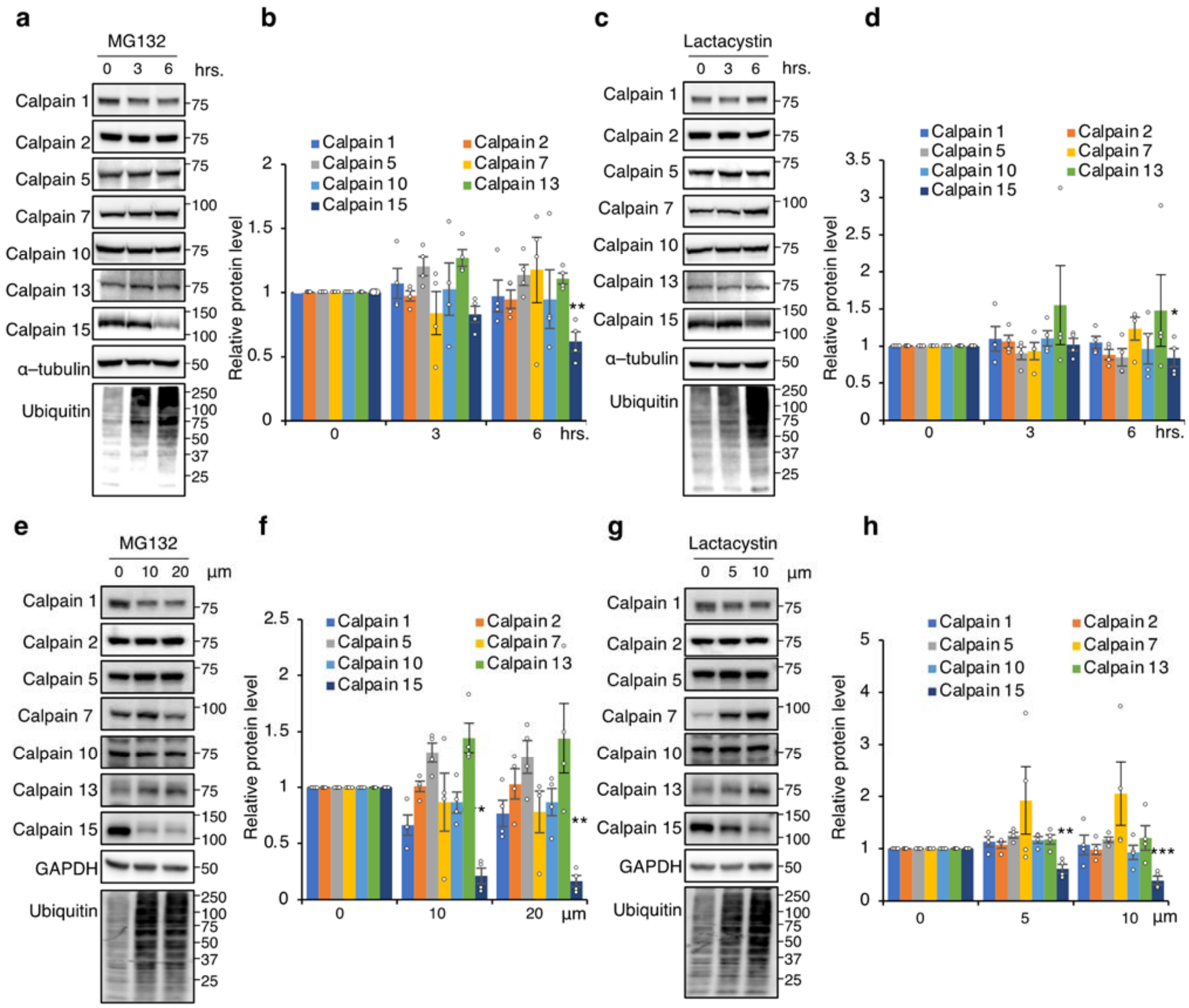

Fig. S4: Calpains are regulated via ubiquitin-mediated modification. **a**, **b**, Immunoblot analysis of HEK293 cells treated with MG132 (10 μM) or vehicle using antibodies for ubiquitous expressing calpains. **c**, **d**, Immunoblot analysis of HEK293 cells treated with lactacystin (20 μM) or vehicle using antibodies for ubiquitous expressing calpains. **e**, **f**, Immunoblot analysis of HEK293 cells treated with MG132 or vehicle for 24 h using antibodies for ubiquitous expressing calpains. **g**, **h**, Immunoblot analysis of HEK293 cells treated with lactacystin or vehicle for 24 h using antibodies for ubiquitous expressing calpains. Data represent mean ± SEM, $n = 5$; *$P < 0.05$, **$P < 0.01$, ***$P < 0.0001$, ns = not significant.

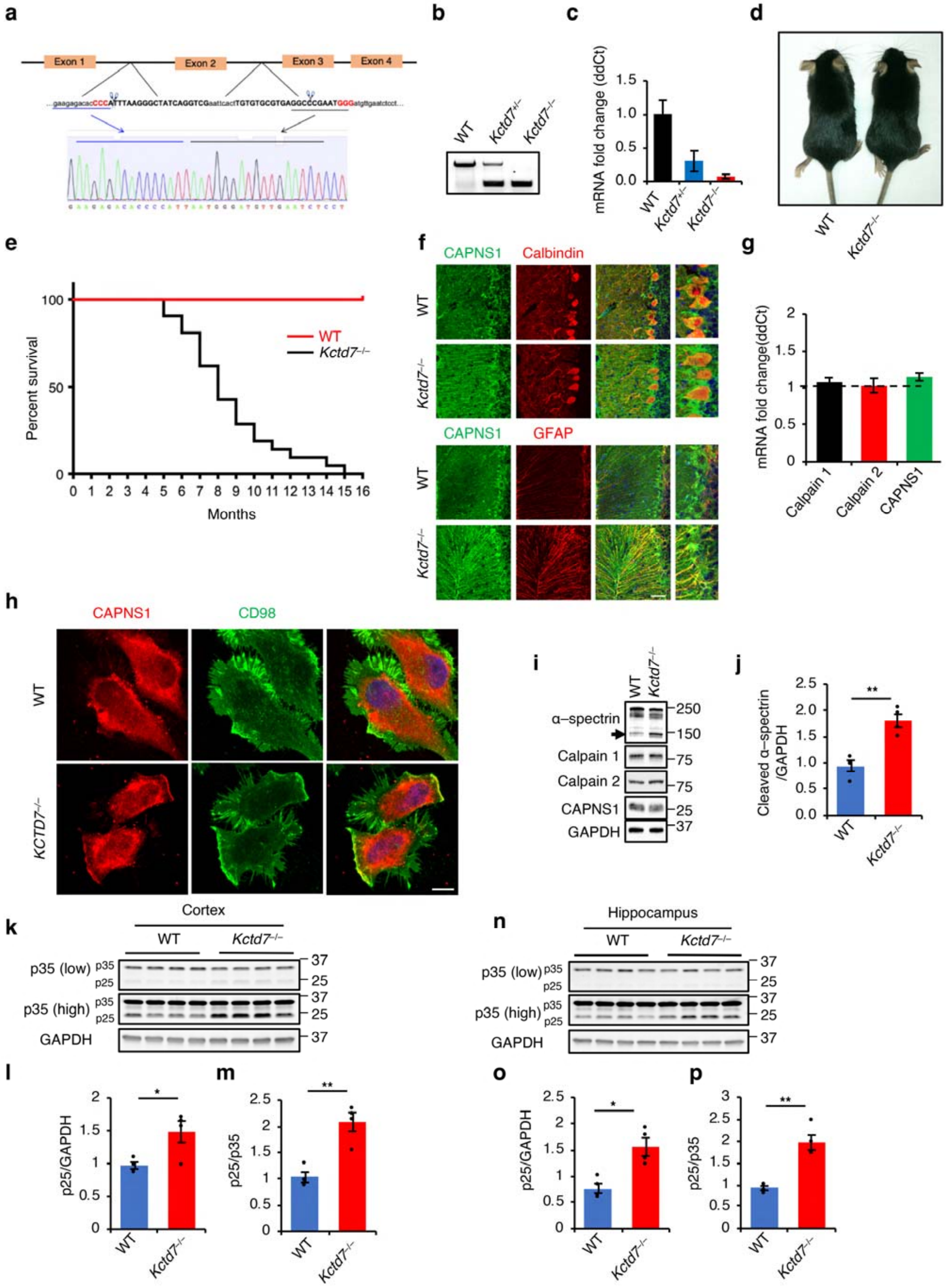

a
Exon 1
Exon 2
Exon 3
Exon 4
b
WT
Kctd7+/-
Kctd7-/-
c
mRNA fold change (ddCt)
d
e
Percent survival
Months
WT
Kctd7-/-
f
CAPNS1
Calbindin
GFAP
g
mRNA fold change(ddCt)
Calpain 1
Calpain 2
CAPNS1
h
CD98
KCTD7-/-
i
α-spectrin
GAPDH
j
Cleaved α-spectrin /GAPDH
k
Cortex
p35 (low)
p35 (high)
p35
p25
n
Hippocampus
l
p25/GAPDH
m
p25/p35
o
p

Fig. S5: Generation of a *Kctd7*$^{-/-}$ mouse line using CRISPR/Cas9 technology. a, Two gRNAs were designed to cut both ends of exon 2 of the *KCTD7* gene. **b**, Agarose gel electrophoresis analysis of PCR products using genomic DNA from a WT mouse and heterozygous (*Kctd7*$^{+/-}$) and homozygous (*Kctd7*$^{-/-}$) mice from the selected *Kctd7*$^{-/-}$ mouse line. **c**, RT-qPCR analysis of mRNAs extracted from WT, *Kctd7*$^{+/-}$ and *Kctd7*$^{-/-}$ mouse brain homogenates showing transcriptional levels of *Kctd7* (*n* = 3 per genotype). **d**, Representative pictures of 5-week-old WT and *Kctd7*$^{-/-}$ mice. **e**, Survival curve of *Kctd7*$^{-/-}$ mice (median lifespan = 9 months; *n* = 22 for WT mice, *n* = 20 for *Kctd7*$^{-/-}$ mice). **f,** Confocal microscopy of 5-week-old WT and *Kctd7*$^{-/-}$ mouse cerebellum showing CAPNS1 localization in Purkinje cells and astrocytes. α-calbindin and α-GFAP antibodies were used to mark Purkinje cells and astrocytes, respectively. **g**, RT-qPCR analysis of mRNAs extracted from 5-week-old WT and *Kctd7*$^{-/-}$ mouse brain homogenates showing no differences in transcriptional levels of calpain 1, calpain 2, and CAPNS1 (*n* = 3 per genotype). **h,** Confocal microscopy analysis of CAPNS1 localization in WT or *KCTD7* knock-out HeLa cells. CD98 was used as a plasma membrane marker. Scale bar, 10 μm. **i**, **j**, Four-week-old astrocyte cultures were lysed in RIPA buffer and α-spectrin cleavage was evaluated by immunoblotting. **k-p**, Cortical and hippocampal lysates from 5-week-old WT and *Kctd7*$^{-/-}$ mice were lysed in NP-40 lysis buffer and immunoblotted for p35 to evaluate calpain-mediated cleavage. Data represent mean ± SEM, (*n* = 4 per genotype); $^{*}P < 0.05$, $^{**}P < 0.01$, $^{***}P < 0.0001$, ns = not significant.

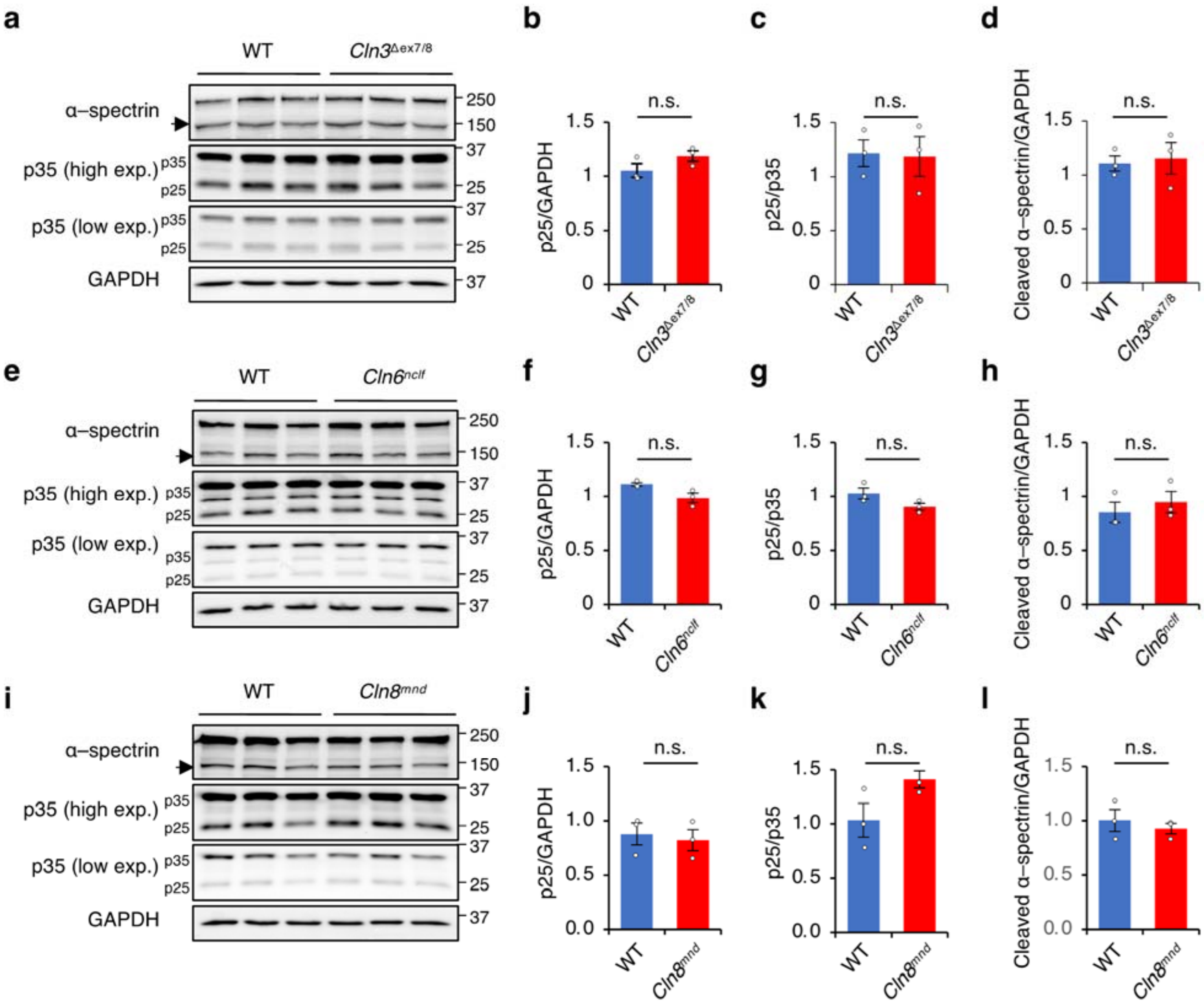

Fig. S6: Analysis of calpain activity in various mouse models of NCL. a-l, Calpain activity was evaluated in *Cln3*$^{\Delta ex7/8}$ mice (a-d), *Cln6*^{nclf} mice (e-h), and *Cln8*^{mnd} mice (i-l) by performing immunoblot analysis of whole brain lysates collected at 2-3 months of age with antibodies for α-spectrin and p35. GAPDH was used as a loading control. n = 3 per genotype. Data represent mean ± SEM; $^{*}P < 0.05$, $^{**}P < 0.01$, $^{***}P < 0.0001$, ns = not significant.

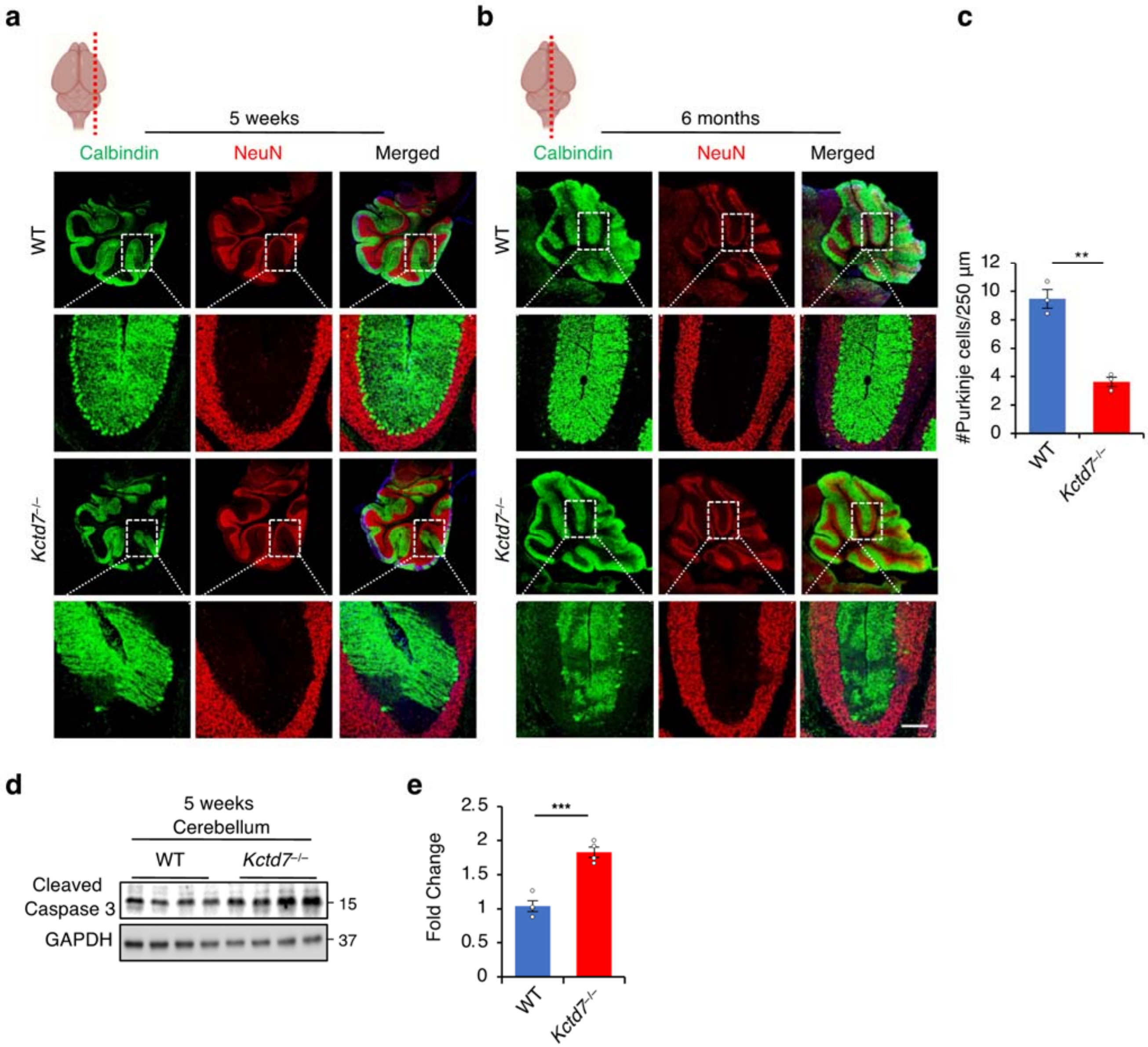

Fig. S7: KCTD7 deficiency leads to neurodegeneration. a-c, Confocal microscopy analysis of the cerebellum from 5-week-old and 6-month-old WT and *Kctd7*$^{-/-}$ mice. α-calbindin antibody was used to label Purkinje cells. Scale bars, 200 µm. Quantification of Purkinje cell numbers in 6-month-old animals shown in (c), $n = 5$ per genotype. Scale bars, 200 µm. **d**, **e**, Immunoblot analysis of cerebellar tissue from 5-week-old WT and *Kctd7*$^{-/-}$ mice using cleaved caspase 3 antibody. Data represent mean ± SEM, $n = 4$; $^{*}P < 0.05$, $^{**}P < 0.01$, $^{***}P < 0.0001$, ns = not significant.

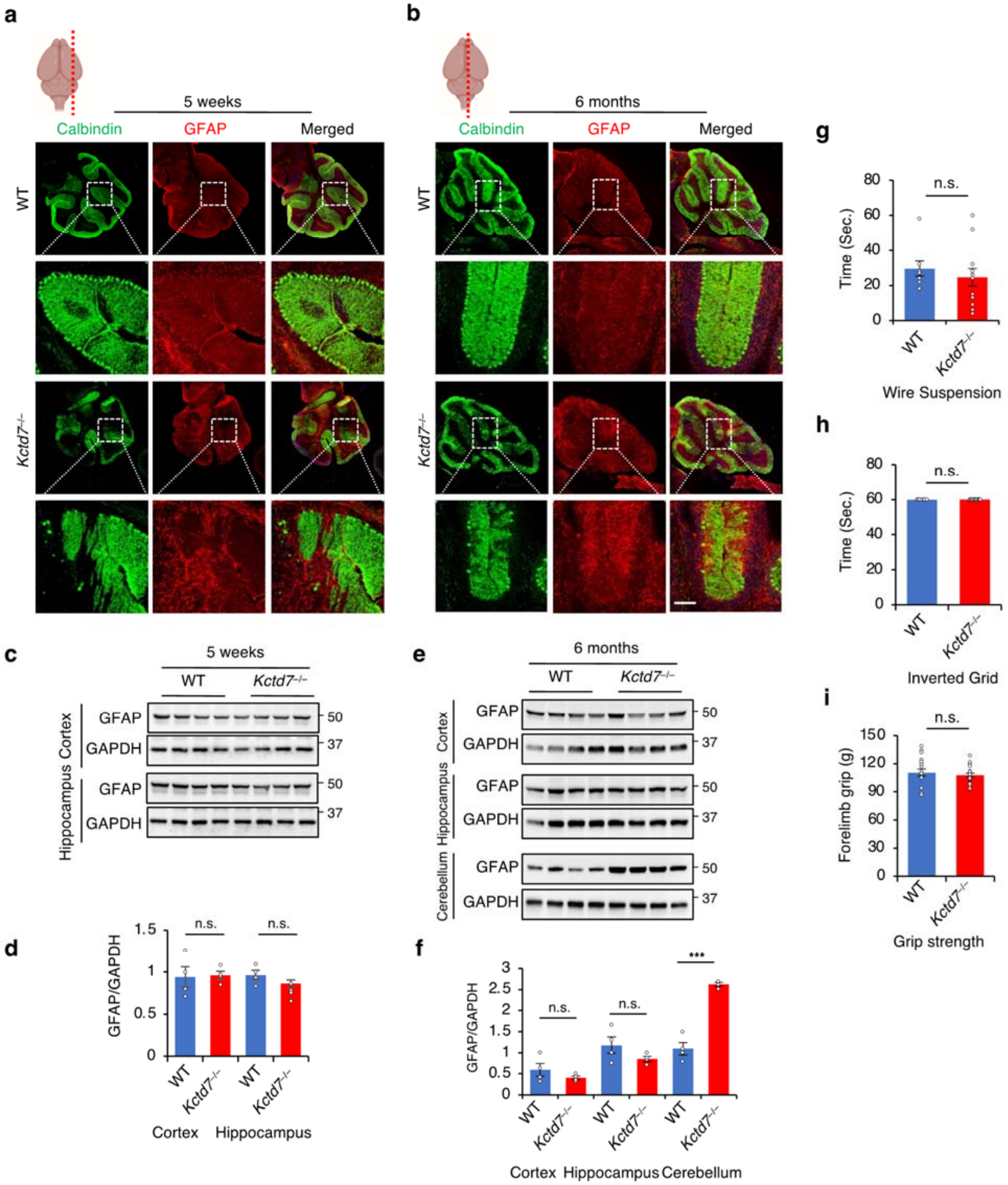

Fig. S8: KCTD7 deficiency leads to neurodegeneration and behavioral impairments. a, **b**, Confocal microscopy analysis of the cerebellum from 5-week-old and 6-month-old WT and *Kctd7*$^{-/-}$ mice. α-GFAP antibody was used to label astrocytes. **c**, **d**, Immunoblot analysis of

cortical and hippocampal tissue from 5-week-old WT and *Kctd7*$^{-/-}$ mice using α-GFAP antibody. GAPDH is used as a loading control. **e**, **f**, Immunoblot analysis of cortical, hippocampal, and cerebellar tissue from 6-month-old WT and *Kctd7*$^{-/-}$ mice using α-GFAP antibody. GAPDH is used as a loading control. **g**, **h**, WT and *Kctd7*$^{-/-}$ mice were assessed for latency to fall in a wire suspension test (g) and inverted grid tests (h) at 10 weeks of age. n = 10 for WT mice; n = 16 for *Kctd7*$^{-/-}$ mice. **i**, WT and *Kctd7*$^{-/-}$ mice were assessed for grip strength in a forepaw grip strength at 10 weeks of age using a digital force gauge. n = 10 for WT mice; n = 16 for *Kctd7*$^{-/-}$ mice. Data represent mean ± SEM. $^{*}P < 0.05$, $^{**}P < 0.01$, $^{***}P < 0.0001$, ns = not significant.

Supplementary Table 1. List of KCTD7-interacting proteins as detected by LC-MS/MS.

Accession No.	Name	No. Peptides
	Replicate #1	
gi|825635	calmodulin	634
gi|62897129	heat shock 70kDa protein 8 isoform 1 variant	143
gi|18088719	Tubulin, beta	102
gi|269784642	BTB/POZ domain-containing protein KCTD7 isoform 2	82
gi|297691739	Tubulin alpha-1B chain isoform 2	51
gi|111305821	Valosin-containing protein	19
gi|187281	M4 protein	19
gi|4502565	calpain small subunit 1	48
gi|302129652	annexin A6 isoform 2	19
gi|20149594	heat shock protein HSP 90-beta	17
gi|62897075	heat shock 70kDa protein 9B precursor variant	17
gi|3139079	cullin 3	10
gi|47115317	VIM	8
gi|6682361	talin	9
gi|119619622	reticulocalbin 2, EF-hand calcium binding domain	8
gi|375314781	keratin 1	8
gi|28277071	Importin 5	11
gi|5107666	Chain A, Structure Of Importin Beta Bound To The Ibb Domain Of Importin Alpha	8
gi|19111150	angiomotin isoform 2	8
gi|48145697	CPNE1	8
gi|62897625	beta actin variant	8
gi|49168528	ANXA5	9
gi|62898293	programmed cell death 6 variant	6
gi|5734603	KARP-1-binding protein 2 (KAB2)	6
gi|31221	protein serine/threonine kinase	8
gi|38571606	Ribosomal protein L23	7
gi|9963810	NRAGE	5
gi|662841	heat shock protein 27	12
gi|48145673	HNRPH1	6
gi|73915357	Protein transport protein Sec16A;	5
gi|12017959	C1orf28	6
gi|49065418	BAG2	9
gi|119596821	myosin light chain kinase 2, skeletal muscle, isoform CRA_b	13
gi|68533039	DSP variant protein	4
gi|31544959	TRIM28 protein	4
gi|40788339	KIAA0723 protein	4

gi|62089432	DnaJ (Hsp40) homolog, subfamily A, member 3 variant	4
gi|78042577	MAP7 domain-containing protein 1	3
gi|158256510	unnamed protein product	5
gi|62089254	DnaJ (Hsp40) homolog, subfamily C, member 7 variant	3
gi|18105007	CAD protein	4
gi|62898934	DnaJ (Hsp40) homolog, subfamily B, member 6 isoform a variant	3
gi|56122599	leukemia multidrug resistance associated protein	4
gi|187609788	truncated plakophilin-2	3
gi|119617041	keratin 5 (epidermolysis bullosa simplex, Dowling-Meara/Kobner/Weber-Cockayne types), isoform CRA_d	3
gi|8925970	eIF4E-transporter	3
gi|49168458	DNAJB1	3
gi|904032	p48	3
gi|119600190	ribosomal protein L24, isoform CRA_e	4
gi|9246427	RNA helicase A binding protein 95	3
gi|88759348	RING finger protein 219	4
gi|63252886	prolyl 4-hydroxylase subunit alpha-1 isoform 1 precursor	2
gi|310750364	ensconsin isoform 6	2
gi|62896517	ribosomal protein S4, X-linked X isoform variant	2
gi|62088004	EGF-containing fibulin-like extracellular matrix protein 1 isoform b variant	2
gi|790817	microfibril-associated glycoprotein 4, partial	4
gi|62897071	oxygen regulated protein precursor variant	2
gi|453155	keratin 9	2
gi|62896605	eukaryotic translation elongation factor 1 alpha 1 variant	2
gi|169159111	tubulin tyrosine ligase-like family, member 12	2
gi|8394343	sperm surface protein Sp17	2
gi|62896539	chaperonin containing TCP1, subunit 8 (theta) variant	2
gi|71891685	KIAA0829 protein	2
gi|340026	tyrosine kinase, partial	2
gi|253735647	HsMcm7	2
gi|3088356	ribosomal protein L38	2
gi|71533987	General transcription factor II, i	2
gi|197692147	T-complex protein 1 subunit beta	2
gi|42476299	PERQ amino acid-rich with GYF domain-containing protein 2 isoform b	3
gi|23242517	Copine III	4
gi|317373596	Calpain-2 catalytic subunit	2
gi|12804313	Similar to expressed sequence 2 embryonic lethal, partial	2
gi|397524998	Tescalcin isoform 1	2
gi|61656607	tumor rejection antigen (gp96) 1	3
gi|45709422	Karyopherin alpha 2 (RAG cohort 1, importin alpha 1)	2
gi|5031579	A-kinase anchor protein 8	2

gi|495126	ribosomal protein L11	2
gi|194390416	eukaryotic translation initiation factor 3, subunit 7 zeta, 66/67kDa, isoform CRA_b	2
gi|119623811	hCG2005638, isoform CRA_c	2
gi|452269	glucose-6-phosphate dehydrogenase	2
gi|194374093	unnamed protein product	2
gi|8099630	protein kinase CDK9	3
gi|49456297	PRDX4	2
gi|13661193	fibulin-1 isoform D precursor	2
gi|15147337	E3 ubiquitin-protein ligase UBR5	2
gi|15667255	calpain small subunit 2	2
gi|643016	T cell receptor alpha chain Mb11	2
	Replicate #2	
gi|825635	calmodulin	267
gi|62897129	heat shock 70kDa protein 8 isoform 1 variant	54
gi|27368062	class IVb beta tubulin	44
gi|269784642	BTB/POZ domain-containing protein KCTD7 isoform 2	68
gi|375314781	keratin 1	24
gi|297691739	tubulin alpha-1B chain isoform 2	21
gi|119581085	keratin 10 (epidermolytic hyperkeratosis; keratosis palmaris et plantaris), isoform CRA_b	14
gi|28277071	Importin 5	14
gi|20149594	heat shock protein HSP 90-beta	15
gi|5107666	Chain A, Structure Of Importin Beta Bound To The Ibb Domain Of Importin Alpha	13
gi|302129652	annexin A6 isoform 2	18
gi|4502565	calpain small subunit 1	22
gi|89059026	extracellular signal-regulated kinase-2 splice variant	11
gi|62898934	DnaJ (Hsp40) homolog, subfamily B, member 6 isoform a variant	7
gi|15277503	ACTB protein, partial	8
gi|197101391	40S ribosomal protein S3	5
gi|48734966	Eukaryotic translation elongation factor 1 alpha 1	7
gi|49065418	BAG2	7
gi|14141152	heterogeneous nuclear ribonucleoprotein M isoform a	5
gi|119611832	hCG2027326	6
gi|150439331	copine I	5
gi|47115317	VIM	6
gi|435476	cytokeratin 9	7
gi|62088004	EGF-containing fibulin-like extracellular matrix protein 1 isoform b variant	5
gi|18105007	CAD protein	5
gi|119613655	calpain 2, (m/II) large subunit, isoform CRA_a	5
gi|62897075	heat shock 70kDa protein 9B precursor variant	6

gi|66365795	Histone cluster 1, H1e	4
gi|15126735	Heat shock 27kDa protein 1	5
gi|20521736	KIAA1027 protein	4
gi|119571370	hCG1985580, isoform CRA_a	3
gi|3139079	cullin 3	3
gi|15080163	DnaJ (Hsp40) homolog, subfamily A, member 3	3
gi|38571606	Ribosomal protein L23	3
gi|49456627	CALU	3
gi|3088356	ribosomal protein L38	3
gi|62089254	DnaJ (Hsp40) homolog, subfamily C, member 7 variant	3
gi|1916622	HAX-1	4
gi|17391477	Annexin A5	3
gi|119600195	hCG1777176, isoform CRA_b	2
gi|119618532	hCG2016250, isoform CRA_a	2
gi|60299991	aging-associated protein 14b	3
gi|14043538	MYLK2 protein	6
gi|23242517	Copine III	4
gi|194389120	unnamed protein product	2
gi|158260761	unnamed protein product	3
gi|119619622	reticulocalbin 2, EF-hand calcium binding domain	2
gi|5174661	protein S100-A2	2
gi|31418053	CCT8 protein	2
gi|116283604	SPA17 protein	2
gi|114306812	BAT1 protein	2
gi|111305821	Valosin-containing protein	2
gi|109389365	glucose-6-phosphate 1-dehydrogenase isoform a	3
gi|386803	40-kDa keratin protein, partial	3
	Replicate #3	
gi|4529893	HSP70-1	112
gi|66360457	Chain O, Crystal Structure Of Anthrax Edema Factor (Ef) Truncation Mutant, Ef-Delta 64 In Complex With Calmodulin	186
gi|20809886	Tubulin, beta 2C	75
gi|269784642	BTB/POZ domain-containing protein KCTD7 isoform 2	81
gi|111305821	Valosin-containing protein	35
gi|119612249	E3 ubiquitin protein ligase, HECT domain containing, 1, isoform CRA_a	23
gi|297691739	tubulin alpha-1B chain isoform 2	42
gi|3139079	cullin 3	19
gi|1147813	desmoplakin I	23
gi|18105007	CAD protein	20
gi|4502565	calpain small subunit 1	28
gi|49065418	BAG2	14

gi|262206280	dnaJ homolog subfamily C member 7 isoform 1	14
gi|119619622	reticulocalbin 2, EF-hand calcium binding domain	13
gi|19111150	angiomotin isoform 2	11
gi|48734966	Eukaryotic translation elongation factor 1 alpha 1	15
gi|15080163	DnaJ (Hsp40) homolog, subfamily A, member 3	11
gi|14141152	heterogeneous nuclear ribonucleoprotein M isoform a	16
gi|6470150	BiP protein, partial	24
gi|119578931	DnaJ (Hsp40) homolog, subfamily A, member 1, isoform CRA_d	13
gi|62897075	heat shock 70kDa protein 9B precursor variant	20
gi|1699027	nuclear corepressor KAP-1	11
gi|197692465	ATP-dependent RNA helicase DDX5	9
gi|28277071	Importin 5	14
gi|49168458	DNAJB1	9
gi|42406316	FAM29A protein, partial	9
gi|20149594	heat shock protein HSP 90-beta	12
gi|71891790	KIAA0310 protein	9
gi|332808466	40S ribosomal protein S27-like	7
gi|662841	heat shock protein 27	13
gi|197101391	40S ribosomal protein S3	8
gi|47115317	VIM	6
gi|48146259	CCT2	6
gi|55962085	centrosomal protein 170kDa	7
gi|38571606	Ribosomal protein L23	13
gi|20521736	KIAA1027 protein	6
gi|119582464	hCG1982388, isoform CRA_d	8
gi|1916622	HAX-1	5
gi|302129652	annexin A6 isoform 2	12
gi|219519019	5-methyltetrahydrofolate-homocysteine methyltransferase	7
gi|62898934	DnaJ (Hsp40) homolog, subfamily B, member 6 isoform a variant	7
gi|3170178	antigen NY-CO-7	7
gi|119618532	hCG2016250, isoform CRA_a	5
gi|119599729	RuvB-like 1 (E. coli)	5
gi|217272894	116 kDa U5 small nuclear ribonucleoprotein component isoform b	6
gi|119574194	heterogeneous nuclear ribonucleoprotein H1 (H), isoform CRA_b	8
gi|6822170	zinc finger protein	6
gi|31873894	hypothetical protein	9
gi|33112885	acetyl-CoA carboxylase 1	5
gi|150439331	copine I	4
gi|12275876	tripartite motif protein TRIM27 beta	5
gi|49472841	A-kinase anchor protein 8-like	6

gi|15030240	ATP synthase, H+ transporting, mitochondrial F1 complex, alpha subunit 1, cardiac muscle	5
gi|119623458	DiGeorge syndrome critical region gene 14, isoform CRA_a	5
gi|62898013	F-actin capping protein alpha-1 subunit variant	4
gi|385719194	caseinolytic peptidase B protein homolog isoform 2	4
gi|51476906	hypothetical protein	3
gi|5107666	Chain A, Structure Of Importin Beta Bound To The Ibb Domain Of Importin Alpha	4
gi|4337109	BAT3	7
gi|20384797	extracellular signal-related kinase 1c	6
gi|119594339	damage-specific DNA binding protein 1, 127kDa, isoform CRA_a	6
gi|1870688	Bruton's tyrosine kinase-associated protein-135	3
gi|119568002	t-complex 1, isoform CRA_b	5
gi|62896517	ribosomal protein S4, X-linked X isoform variant	7
gi|3088356	ribosomal protein L38	5
gi|68533031	FASN variant protein	3
gi|15277503	ACTB protein, partial	3
gi|119580514	eukaryotic translation initiation factor 3, subunit 7 zeta, 66/67kDa, isoform CRA_b	5
gi|14043538	MYLK2 protein	8
gi|119600993	chromosome 13 open reading frame 7, isoform CRA_b	3
gi|28175596	FARSA protein	3
gi|33150546	D-myo-inositol-3-phosphate synthase	3
gi|119614756	hCG2000485	4
gi|4507677	endoplasmin precursor	5
gi|62131678	14-3-3 protein epsilon isoform transcript variant 1	4
gi|20521013	KIAA0360	4
gi|119583300	melanoma antigen family D, 1, isoform CRA_a	5
gi|56205679	ribosomal protein L5	2
gi|4757732	apoptosis-inducing factor 1, mitochondrial isoform 1 precursor	4
gi|3283882	BDP-1 protein	3
gi|34782987	CAND1 protein, partial	2
gi|38079	75 kDa subunit NADH dehydrogenase precursor	3
gi|197692279	cyclin-dependent kinase 9	3
gi|63102091	AMOT protein, partial	6
gi|55960506	chaperonin containing TCP1, subunit 3 (gamma)	4
gi|119619424	hCG18053	3
gi|40788224	KIAA0355	3
gi|38607488	breast cancer-associated antigen SGA-56M	2
gi|12017959	C1orf28	2
gi|21619217	C1orf142 protein	2
gi|157311635	protein FAM83H	3

gi|17391477	Annexin A5	5
gi|119608918	plakophilin 2, isoform CRA_b	2
gi|2150046	26S proteasome subunit 9	2
gi|1620018	heat shock protein 90	2
gi|14424674	CDC42 effector protein (Rho GTPase binding) 1	3
gi|119627021	hook homolog 1 (Drosophila), isoform CRA_b	2
gi|119588620	glutamine and serine rich 1	3
gi|49456627	CALU	2
gi|31418053	CCT8 protein	2
gi|189306	nucleolin	2
gi|119584552	IQ motif and Sec7 domain 1, isoform CRA_b	2
gi|114432124	activating molecule in beclin-1-regulated autophagy	2
gi|1890050	cysteine protease	2
gi|19851923	CLL-associated antigen KW-14	2
gi|13111995	Ubiquitin associated protein 2-like	2
gi|11967975	myeloid leukemia factor 1 isoform 1	2
gi|119600195	hCG1777176, isoform CRA_b	2
gi|291868	ATPase	2
gi|119610743	complement component 1, q subcomponent binding protein, isoform CRA_c	2
gi|5802968	protein AF1q	3
gi|187957150	KIAA0841	2
gi|40807091	GANAB protein	2
gi|306890	chaperonin (HSP60)	3
gi|119617721	nucleosome assembly protein 1-like 1, isoform CRA_b	3
gi|3005758	RNA polymerase II 140 kDa subunit	2
gi|330864767	dnaJ homolog subfamily B member 5 isoform 2	5
gi|62089430	DnaJ (Hsp40) homolog, subfamily B, member 4 variant	2
gi|23270929	DEAD (Asp-Glu-Ala-Asp) box polypeptide 20	2
gi|758423	zinc-finger protein	3
gi|124001558	inactive ubiquitin carboxyl-terminal hydrolase 54	2
gi|58257735	KIAA0651 protein	3
gi|2209374	HsCdc7	2
gi|119620390	chaperonin containing TCP1, subunit 4 (delta), isoform CRA_a	2
gi|332836269	reticulocalbin-1	2
gi|5771523	3-phosphoglycerate dehydrogenase	2
gi|3170190	antigen NY-CO-25	2
gi|8885790	filamin 2	3
gi|197692141	ATP-dependent RNA helicase DDX3X	3
gi|26051235	nuclear pore complex protein Nup133	3
gi|207029415	histone-binding protein RBBP4 isoform b	2

gi|214829673	ADP-ribosylation factor GTPase-activating protein 3 isoform 1	2
gi|226342869	TBC1 domain family member 15 isoform 2	2
gi|15126760	Proteasome (prosome, macropain) 26S subunit, non-ATPase, 3	3
gi|33869643	EEF2 protein, partial	2
gi|119610102	procollagen-proline, 2-oxoglutarate 4-dioxygenase (proline 4-hydroxylase), beta polypeptide, isoform CRA_d	2
gi|62088004	EGF-containing fibulin-like extracellular matrix protein 1 isoform b variant	3
gi|109389365	glucose-6-phosphate 1-dehydrogenase isoform a	2
gi|39963074	PRP8 pre-mRNA processing factor 8 homolog (S. cerevisiae)	2
gi|58761504	HAUS augmin-like complex subunit 8 isoform a	3
gi|52545638	hypothetical protein	3
gi|119570975	ubiquinol-cytochrome c reductase core protein II, isoform CRA_a	2
gi|397524998	tescalcin isoform 1	4
gi|226246671	40S ribosomal protein S20 isoform 1	2
gi|103471999	serine/threonine-protein kinase 3 isoform 1	2
gi|553640	ribosomal protein S13	2
gi|119570641	hCG1994130, isoform CRA_b	2
gi|119611476	centrosomal protein 350kDa, isoform CRA_a	2
gi|5689435	KIAA1049 protein	2
gi|20067381	ALMS1 protein	3
gi|62897195	glutamate dehydrogenase 1 variant	2
gi|16876910	HNRPF protein	7
gi|4885079	ATP synthase subunit gamma, mitochondrial isoform H (heart) precursor	2
gi|48146309	DNAJB11	2
gi|51094698	MAD1 mitotic arrest deficient-like 1 (yeast)	2
gi|50949411	hypothetical protein	2
gi|119617974	PCTAIRE protein kinase 2, isoform CRA_c	2
gi|47938913	HYOU1 protein	2
gi|119608880	hCG1979429, isoform CRA_a	2
gi|62548856	BAG family molecular chaperone regulator 5 isoform a	2
gi|23242517	Copine III	3
gi|14017863	KIAA1823 protein	2
gi|45861372	200 kDa U5 snRNP-specific spliceosomal protein	2
gi|2370178	P73	2
gi|395132436	60S ribosomal protein L18 isoform 2	2
gi|21280444	AF15q14	2
gi|1419564	cytokeratin	2
gi|156181166	DnaJ	4
gi|2078529	Hlark	2
gi|180928	core protein II precursor	2
gi|89059026	extracellular signal-regulated kinase-2 splice variant	4

gi|19353009	Similar to Elongation factor 2b, partial	2
gi|3170190	antigen NY-CO-25	2

Supplementary Table S2. Putative target sites of ubiquitination in CAPN1 and CAPN2 as calculated by UbiNet.

Serial Number	Amino Acid	Probability Score	Ubiquitination site
		Calpain 1	
1	461	0.99849	QPAVHL K RDFFLA
2	36	0.664366	RHENAI K YLGQDY
3	398	0.634094	WVNPQF K IRLDET
4	564	0.998944	DMEISV K ELRTIL
5	631	0.601169	RKFDLD K SGSMSA
6	71	0.710379	PQSLGY K DLGPNS
7	365	0.998614	KSRTIR K WNTTLY
8	193	0.515752	WSALLE K AYAKVN
9	197	0.510909	LEKAYA K VNGSYE
10	20	0.577844	VSAQVQ K QRAREL
11	626	0.659654	YLSIFR K FDLDKS
12	171	0.608432	DDLLPI K DGKLVF
13	240	0.558482	LYQIIL K ALERGS
14	359	0.595199	LTPDAL K SRTIRK
15	328	0.582712	RDQLRV K MEDGEF
16	280	0.520307	YSVTGA K QVNYRG
		Calpain 2	
1	7	0.56294	MAGIAA K LAKDRE
2	10	0.572412	IAAKLA K DREAAE
3	26	0.998295	SHDRAI K YLNQDY
4	61	0.582818	PSALGF K ELGPYS
5	183	0.52848	WSALLE K AYAKIN
6	280	0.532409	SNGSLQ K LIRIRN
7	476	0.545334	EVLNRF K LPPGEY
8	629	0.512513	NSYEMR K ALEEAG
9	674	0.998452	RLETLF K IFKQLD
10	677	0.661898	TLFKIF K QLDPEN

Supplementary Table S3. List of primary and secondary antibodies.

Antibody	Supplier	Catalog No.	Dilution
Primary Antibodies			
Anti-Rabbit CAPN1	Cell Signaling	2556	WB 1:1000
Anti-Rabbit CAPN2	Cell Signaling	2539	WB 1:1000
Anti-Rabbit CAPNS1	Sigma	HPA006872	WB 1:1000
Anti-Rabbit CAPNS1	Sigma	HPA006872	IF 1:250
Anti-Mouse CAPN1	Santa Cruz	sc-390677	WB 1:250
Anti-Mouse CAPN1	Sigma	C0355	WB 1:1000
Anti-Mouse CAPN2	Santa Cruz	sc-373967	WB 1:1000
Anti-Mouse CAPNS1	Santa Cruz	sc-32325	WB 1:500
Anti-Mouse alpha spectrin	Millipore	MAB1622	WB 1:2000
Anti-Rabbit p35	Cell Signaling	2680	WB 1:1000
Anti-Rabbit c-Myc	Sigma	C3956	WB 1:5000
Anti-Rabbit FLAG	Sigma	F7425	WB 1:5000
Anti-Mouse FLAG	Sigma	F1804	WB 1:5000
Anti-Rabbit Cullin 3	Abcam	ab108407	WB 1:500
Anti-Mouse HA	BioLegend	901514	WB 1:5000
Anti-Rabbit KCTD7	ProSci Inc.	61-770	WB 1:500
Anti-Mouse GAPDH	Proteintech	60004-1-Ig	WB 1:20000
Anti-Mouse GFAP	Abcam	ab7260	WB 1:10000
Anti-Mouse GFAP	Sigma	G6171	IF 1:500
Anti-Rabbit HA-Tag (C29F4)	Cell Signaling	3724S	WB 1:1000
Anti-Rabbit Sodium Potassium ATPase	Abcam	ab76020	WB 1:1000
Anti-Rabbit Calreticulin	Cell Signaling	12238	WB 1:1000
Anti-Rabbit Tom20	Santa Cruz	sc-11415	WB 1:1000
Anti-Mouse Calbindin	Swant	300	IF 1:5000
Anti-Mouse Calbindin	Swant	CB38	IF 1:5000
Anti-Rabbit GFP (D5.1)	Cell Signaling	2956S	WB 1:5000
Anti-Rabbit GFP (D5.1)	Cell Signaling	2956S	IF 1:1000
Anti-Rabbit Ubiquitin	Cell Signaling	3933	WB 1:1000
Anti-Rabbit LAMP1 (D2D11)	Cell Signaling	9091	IF 1:250
Anti-Mouse NeuN	Millipore	MAB377	IF 1:500
Secondary Antibodies			
Alexa Fluor® 488 Anti-Rat IgG	Invitrogen	A-11006	1:1000
Alexa Fluor® 488 Anti-Chicken IgG	Invitrogen	A-11039	1:1000
Alexa Fluor® 488 Anti-Mouse IgG	Invitrogen	A-21200	1:1000
Alexa Fluor® 594 Anti-Mouse IgG	Invitrogen	A-21201	1:1000
Alexa Fluor® 594 Anti-Rabbit IgG	Invitrogen	A-21207	1:1000

Alexa Fluor® 633 Anti-Mouse IgG	Invitrogen	A-21052	1:1000
Alexa Fluor® 633 Anti-Goat IgG	Invitrogen	A-21082	1:1000
ECL anti-rabbit IgG-HRP	GE Healthcare	NA9340-1ML	1:5000
ECL anti-chicken IgY-HRP	Santa Cruz	sc-2428	1:5000
ECL anti-goat IgG-HRP	Santa Cruz	sc-2020	1:5000
ECL anti-rat IgG-HRP	GE Healthcare	NA9350-1ML	1:5000
ECL anti-mouse IgG-HRP	GE Healthcare	NA9310-1ML	1:5000

Supplementary Table S4. Oligos used in this study.

Primer name	Forward Primer	Reverse Primer
	Cloning and site-directed mutagenesis	
KCTD7 WT	gccgcgaattcaccatggtggtagtcacgggg	ccggggtaccgcccaccatgtgatcttg
KCTD7 R84W	ccatgttcagtgggtggcactacatcccc	ggggatgtagtgccacccactgaacatgg
KCTD7 R94W	cggactccgagggctggtacttcatcgac	gtcgatgaagtaccagccctcggagtccg
KCTD7 L108M	cacactttggagatgtgatgaatttcctgcgctca	tgagcgcaggaaattcatcacatctccaaagtgtg
KCTD7 D115Y	cctgcgctcagggtatctcccacccaggg	ccctgggtgggagataccctgagcgcagg
KCTD7 R184C	ccagcggaaggcctgctttgccaagct	agcttggcaaagcaggccttccgctgg
CAPNS1	cgactctagaggatccatgttcctggttaactcgtt	agtcagcccgggatccggaatacatagtcagctgc
	Mouse genotyping	
mKCTD7_F1	aacatctgtggccaaaaagc	
mKCTD7_R1	gctggacttccagaggacac	
mKCTD7_F2	cttggacgtgagatggaggg	
mKCTD7_R2	ccaaagtgtgtgccatctcgatcg	
	qRT-PCR	
Cyclophilin	ggcaaatgctggaccaaacacaa	gtaaaatgcccgcaagtcaaaag
CAPN1	gctggccctcatgcagaaac	cgagggacctggtacactgc
CAPN2	gagaggagggaagggaggga	ggaggagccctctcctgaca
CAPNS1	gaatgtgctcggaggcctga	cctcgctgatggcgctaatg
KCTD7	gagaacatgcagccactgaa	tccgctccaagtggtcttt
